# Physiological MplW514L expression in hematopoietic stem cell causes an essential thrombocythemia and progressive myelofibrosis

**DOI:** 10.1172/JCI199690

**Published:** 2026-04-23

**Authors:** Shujing Zhang, Jingjing Liu, Yuan Li, Yi Wang, Lingling Wang, Miaomiao Xu, Yanxia Li, Ge Dong, Shanshan Wang, Yanmei Li, Zhigang Cai, Baobing Zhao

**Affiliations:** 1 State Key Laboratory of Discovery and Utilization of Functional Components in Traditional Chinese Medicine,; 2 Key Laboratory of Chemical Biology (Ministry of Education), School of Pharmaceutical Sciences,; 3 NMPA Key Laboratory for Technology Research and Evaluation of Drug Products, School of Pharmaceutical Sciences, and; 4 Department of Pharmacology, School of Pharmaceutical Sciences, Cheeloo College of Medicine, Shandong University, Jinan, China.; 5 State Key Laboratory of Experimental Hematology, Tianjin Key Laboratory of Inflammatory Biology, Department of Pharmacology, School of Basic Medical Science, Tianjin Medical University, Tianjin, China.; 6 The Key Laboratory of Chemistry for Natural Products of Guizhou Province and Chinese Academic of Sciences, Guiyang, China.

**Keywords:** Hematology, Oncology, Hematopoietic stem cells, Leukemias, Mouse models

## Abstract

Typ515 (W515) mutations in the protein MPL are one of the key driver mutations promoting BCR-ABL-negative myeloproliferative neoplasms (MPNs), but, to our knowledge, their effects on hematopoietic stem cells (HSCs) and MPN-related hematological abnormalities have not been studied in physiological contexts. Here, we established a MplW514L knock-in mouse model, which largely mimics human MPLW515L mutation during hematopoiesis. The mutant mice developed an essential thrombocythemia–like (ET-like) MPN phenotype, displaying excess megakaryopoiesis and thrombocytosis and progressive myelofibrosis. Mechanistically, we observed that the MplW514L-conditioned HSC compartment had a unique disease-initiating capacity; however, it did not exhibit a obvious advantage of competitive repopulation over the WT control. Notably, single-cell analysis and flow cytometry profiles support that MplW514L expression led to a significant expansion of megakaryocyte-biased stem cell fate within the HSC pool. Finally, JAK2 inhibitor treatment phenotypically alleviated the ET signs but failed to eliminate the disease-initiating HSCs. These findings underscore the etiology of physiological expression of the MPLW515L mutation in HSCs and also provide a valuable in vivo model to evaluate potential therapeutic options for patients with MPLW515L-positive MPN.

## Abstract 2

<strong>MPL W515L mutation expands megakaryocyte‑biased HSCs, driving ET‑like MPN that resists JAK inhibitor therapy.</strong>

## Graphical abstract

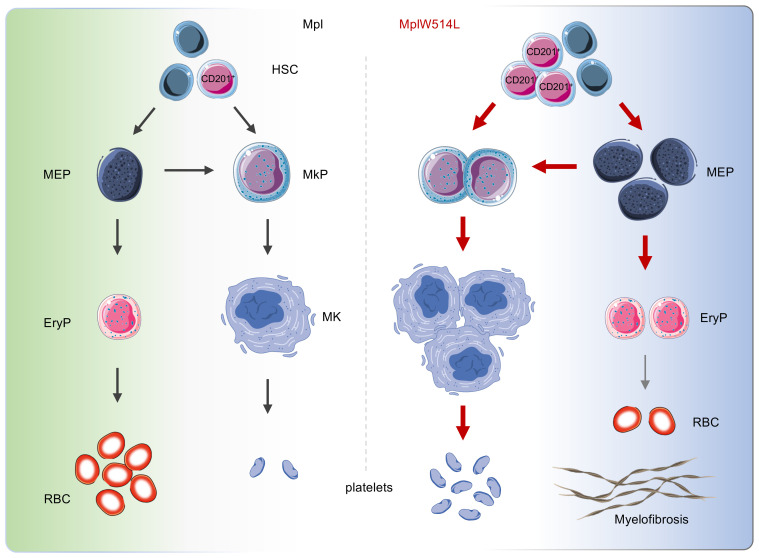

## Introduction

The classical myeloproliferative neoplasms (MPNs), also called BCR-ABL-negative MPNs, include polycythemia vera (PV), essential thrombocythemia (ET), and primary myelofibrosis (PMF) (1). Common molecular events in MPNs are the exclusive mutations in the genes of Janus kinase 2 (JAK2), myeloproliferative leukemia virus oncogene (MPL), or calreticulin (CALR) (2). These somatic mutations are responsible for clonal expansion of HSCs, accompanied by single or multilineage hyperplasia (3). ET is characterized by an increased platelet count with megakaryocytic hyperplasia, whereas PMF is a heterogeneous disorder with clinical and biological characteristics defined by the presence of bone marrow fibrosis and megakaryocytic hyperplasia.

*MPL* (myeloproliferative leukemia virus) is a protooncogene encoding for the receptor of thrombopoietin (TPO). At the physiological level, normal expression of MPL is the primary driver of megakaryocyte differentiation and platelet production (4). However, acquired activating mutations in *MPL* have been found in 5%–10% of patients with PMF and 1%–4% of patients with ET (5). The most frequent mutations are on the tryptophan W515, which is located at the boundary of the transmembrane and the cytosolic domains of the MPL membrane receptor protein, leading to an active dimeric conformation of MPL independent of TPO binding (6, 7). W515L and W515K are the most common mutations in MPNs, while other substitutions are rarely identified, such as W515R, W515A, and W515G (8). More recently, several noncanonical mutations have also been rarely found in MPN that are located in the transmembrane (S505) domain, the extracellular (S204 or E230) or the intracellular (Y591) domains (9, 10). These mutations are usually heterozygous in ET, but can be homozygous during the disease progression to MF (11).

MPLW515L is detectable in CD34^+^ CD38^–^ HSC and mature cell lineages in patients with MPN (12, 13). Overexpression of MPLW515L in cell lines leads to cytokine-independent proliferation and constitutive activation of the JAK/STAT pathway (14, 15). In addition, MPLW515L-positive CD34^+^ cells from patients with MPN showed long-term reconstitution in a nonobese diabetic/severe combined immunodeficient (NOD/SCID) murine model (16). The retroviral *MPLW515L*-transduced model develops an aggressive MPN characterized by marked thrombocytosis, leukocytosis, and bone marrow fibrosis (14). However, the studies fail to identify the disease-initiating hematopoietic compartment(s) and precisely dissect the pathological outcome of the *MplW515L* allele, due to lacking the appropriate models with physiological expression of the MPL variants faithfully mimicking human MPN (17).

Given the central role of activated JAK2 signaling in the pathogenesis of MPNs, JAK2 inhibitors are widely used in the clinic for the treatment of MPNs (18). Although these inhibitors effectively reduce splenomegaly, alleviate MPN-related symptoms, and prolong survival in patients with MPN, they are not able to induce meaningful molecular remissions and reverse the course of the disease in most patients (19, 20). The persistence of disease-initiating cells in quiescent HSCs is the primary reason for relapse and drug resistance (20, 21). Therefore, uncovering the functional characterization of MPLW515L-HSC and its underlying mechanisms for disease-driving cells is essential for providing effective cures for MPL variant–related MPN.

Here, we generated an MplW514L (mimicking human MPLW515L) knock-in mice, in which the expression of MplW514L is under the control of the endogenous *Mpl* promoter. We provided a detailed phenotypic and functional analysis of the MplW514L allele on hematopoietic stem and progenitor cells, and also evaluated the efficacy of JAK2 inhibitors on MPL variant–related MPN in vivo.

## Results

### MplW514L knock-in mice manifested an ET-related MPN phenotype.

**Figure 1:**
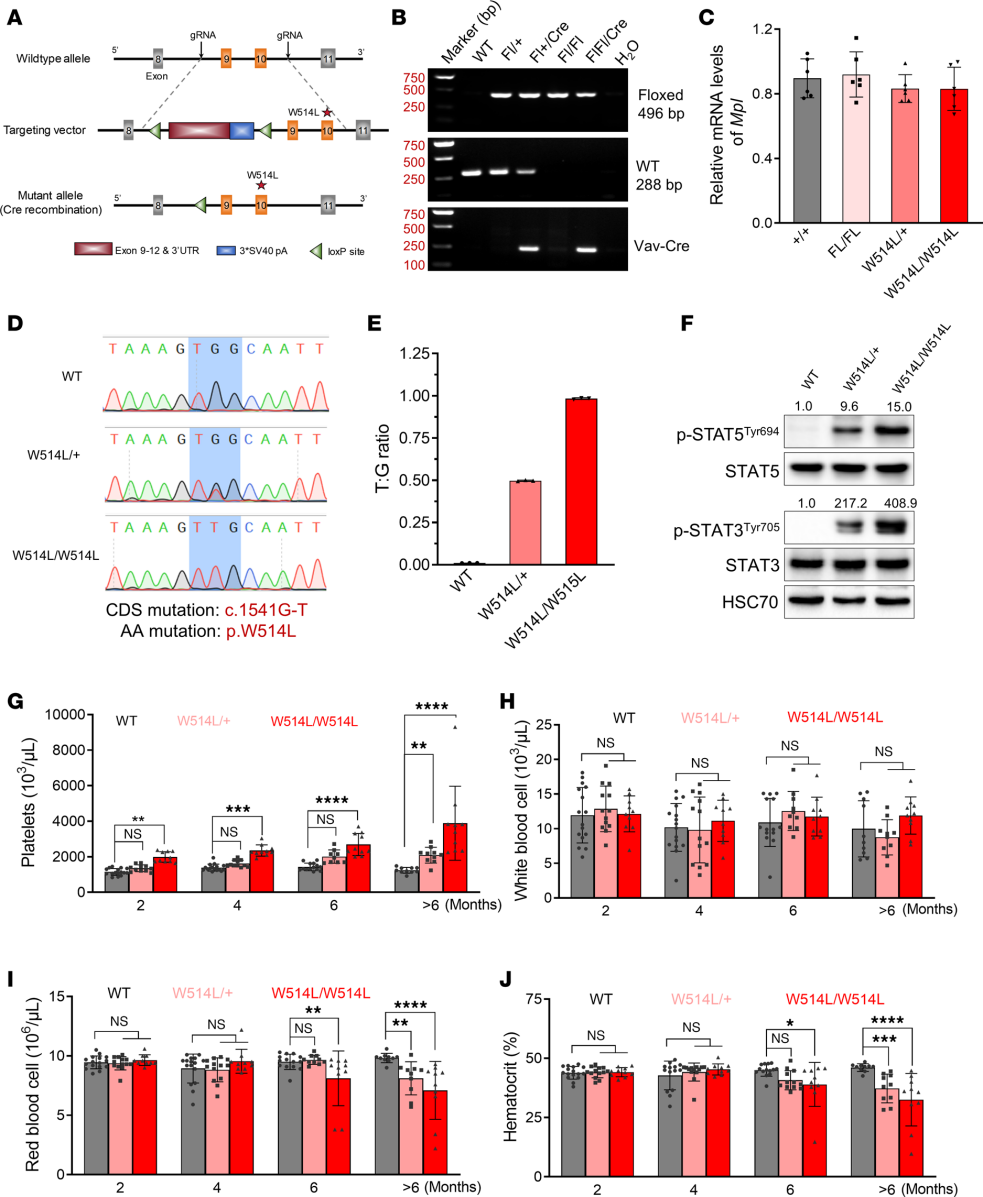
MplW514L knock-in mice manifested an ET-related MPN phenotype. (**A**) Schematic diagram of the gene targeting strategy for MplW514L conditional knock-in mice. (**B**) Genotyping of mice in **A** via PCR using tail DNA. (**C**) Quantitative PCR analysis of *Mpl* mRNA levels in bone marrow mononuclear cells from indicated genotype mice. Data were obtained from 3 different experiments and presented as mean ± SD. (**D**) Chromatogram of sequencing PCR products derived from indicated mice BM cDNA using *Mpl* primers. (**E**) The ratio of MplW514L mutant (T) to the Mpl-WT allele (G) was determined using a standard curve generated by PCR amplification of mixed plasmids encoding MplW514L and Mpl-WT as in Supplemental Figure 1, B and C. Data were presented as mean ± SD, with each dot representing one mouse. (**F**) Immunoblotting analysis of indicated proteins in bone marrow mononuclear cells from indicated genotype mice. HSC70 was used as the loading control. Data were representative of 2 independent experiments. (**G–J**) Peripheral blood parameters of indicated mice at 2–12 months of age. Data were presented as mean ± SD, with each dot representing one mouse. *P* values were determined by 2-way ANOVA with Šidák’s multiple comparisons test. *P < 0.05, **P < 0.01, ***P < 0.001, ****P < 0.0001.

We generated an MplW514L conditional knock-in allele by CRISPR/Cas9-mediated gene engineering in mouse embryonic stem cells, which is the equivalent of the W515L mutation in human MPL. Floxed *MplW514L* mice (FL/FL) were crossed with *Vav-Cre* transgenic mice to induce MplW514L expression controlled by the endogenous *Mpl* promoter in the hematopoietic system (Figure 1A). The overall homology between the mouse and human MPL protein sequences is approximately 80%, with the tryptophan at position 514 in the mouse corresponding to W515 in humans (Supplemental Figure 1A; supplemental material available online with this article; https://doi.org/10.1172/JCI199690DS1). Heterozygous (*FL/+*; *Vav-Cre*, W514L/+) and homozygous (*FL/FL*; *Vav-Cre*, W514L/ W514L) MplW514L-expressing mice were identified by mouse tail PCR (Figure 1B). These mice exhibited similar *Mpl* mRNA levels with WT (*+/+*) littermates, suggesting that the construction strategy targeting W514L mutation did not disrupt the endogenous *Mpl* gene expression (Figure 1C). To determine the frequency of Vav-Cre recombination in MplW514L mice, we sequenced the PCR products derived from BM cDNA of WT, W514L/+, and W514L/W514L mice using *Mpl* primers. The ratio of *MplW514L* mutant to the WT allele was determined using a standard curve generated by PCR amplification of mixed plasmids encoding MplW514L and Mpl (Supplemental Figure 1, B and C). The result confirmed that the recombination efficiency of Vav-Cre was approximately 50% in heterozygous MplW514L-expressing mice and nearly 100% in homozygous MplW514L-expressing mice (Figures 1, D and E). Furthermore, immunoblotting assays revealed a constitutive phosphorylation of STAT5 and STAT3 in MplW514L-expressing BM cells in an allele dose–dependent manner, indicating the hyperactivation of the mutant Mpl protein (Figure 1F).

All mice expressing either heterozygous or homozygous MplW514L exhibited markedly increased platelet counts compared with WT controls at 2 months of age, which is a hallmark feature of ET in humans. Thrombocytosis was more pronounced in homozygous MplW514L-expressing mice and persisted with age (Figure 1G). To assess the long-term hematologic consequences of MplW514L expression, we monitored the mice for up to 48 weeks. Aging MplW514L mice consistently exhibited a sustained ET phenotype (Figure 1G and Supplemental Table 2). Additionally, serum TPO levels in a MplW514L knock-in mice were comparable with those of their WT littermates (Supplemental Figure 1D).

White blood cell and red blood cell counts were not significantly altered in young mice (Figure 1, H–J). Notably, older MplW514L mice (10 months) developed severe anemia, as indicated by significantly reduced RBC counts and hematocrit levels (Figure 1, I and J). In addition, a mild increase in total WBC counts was observed in these older MplW514L mice, primarily due to the elevated neutrophil and monocyte levels (Supplemental Figure 1, E and F), suggesting an age-related shift in myeloid output.

### Aged MplW514L knock-in mice progressed to myelofibrosis.

No marked changes were observed in body weight or spleen weight in young MplW514L mice, although bone marrow cell counts showed a slight increase (Supplemental Figure 2A). However, flow cytometric analysis showed a substantial increase in CD41^+^ cells in the bone marrow of young heterozygous and homozygous MplW514L mice (Supplemental Figure 2B). Histologic analysis also revealed the pronounced megakaryocytic hyperplasia within the MplW514L-expressing bone marrow, characterized by the increased number and size of clustered megakaryocytes (Supplemental Figure 2C).

**Figure 2:**
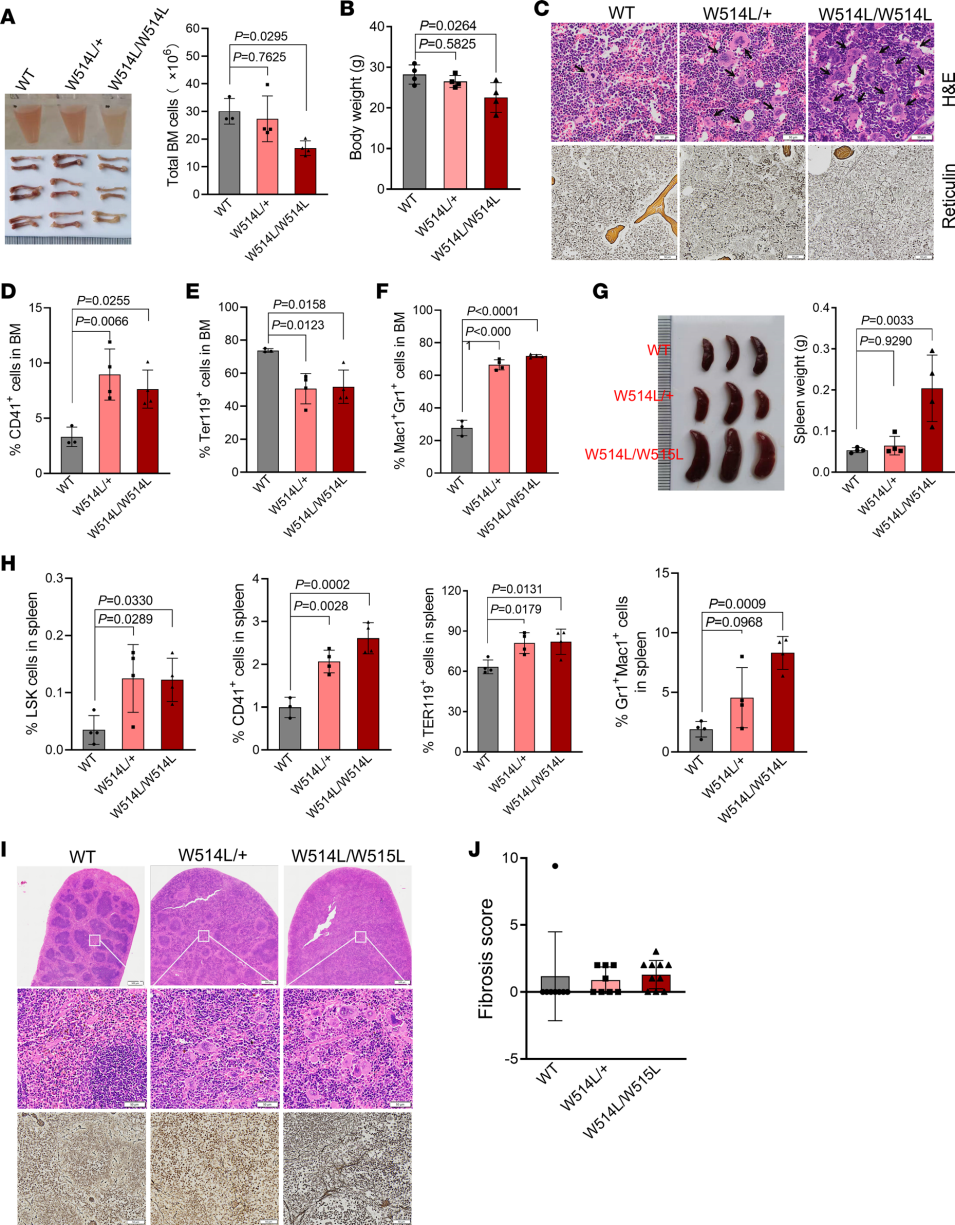
Aged MplW514L mice progressed to myelofibrosis. (**A**) Schematic of mouse femur (left) and Statistical analysis of bone marrow cells (right) in the indicated mice at 10 months of age. Data were presented as mean ± SD, with each dot representing one mouse. (**B**) Statistical analysis of body weight of mice in **A**. Data were presented as mean ± SD, with each dot representing one mouse. (**C**) Representative histopathology of bone marrow from mice as in **A**, including H&E staining (up) and reticulin staining (down). Black arrows indicated the megakaryocytic hyperplasia. Scale bar: 50 μm. (**D**–**F**) Statistical analysis of the proportions of CD41^+^ (**D**), TER119^+^ (**E**), and Mac1^+^Gr1^+^ (**F**) cells from the flow cytometric analysis of bone marrow cell in the indicated mice. Representative flow cytometric pictures were shown in Supplemental Figure 2C. Data were presented as mean ± SD, with each dot representing one mouse. (**G**) Schematic of spleen (left) and statistical analysis of spleen weight (right) in mice in **A**. Each dot represents one mouse. Data were presented as mean ± SD. (**H**) Statistical analysis of the proportions of indicated cells in the mice in **A**. Data were presented as mean ± SD, with each dot representing one mouse. (**I**) Representative histopathology of spleen from mice in **A**, including H&E staining (up) and reticulin staining (down). Scale bar: 50 μm. (**J**) Fibrosis score of aged MplW514L mice (9–12 months). Data shown are representative of 2 independent experiments. All *P* values were determined by 1-way ANOVA with Dunnett’s multiple comparisons test.

Notably, compared with their WT littermates, older MplW514L mice exhibited reduced body weight and bone marrow cell counts (Figure 2, A and B). The major marrow feature of older MplW514L-expressing bone marrow was hyperplasia of the megakaryocytic lineage, accompanied by megakaryocytic atypia and frequent tight clustering of megakaryocytes. Histological examination revealed increased cell size, hyperlobulated or irregularly folded nuclei, and occasional nuclear-cytoplasmic asynchrony, consistent with characteristic features of myelofibrosis (Figure 2C). The frequency of CD41^+^ cells was significantly elevated in the bone marrow of older heterozygous and homozygous MplW514L mice, indicating the sustained megakaryopoiesis and thrombocytosis in these older mice (Figure 2D and Supplemental Figure 2D). Consistent with the reduced RBC levels in the peripheral blood, we found that erythroblasts (TER119^+^) were significantly diminished in the bone marrow of the older MplW514L mice (Figure 2E and Supplemental Figure 2D). In addition, Gr1^+^Mac1^+^ bone marrow cells were also elevated in older heterozygous and homozygous MplW514L-expressing mice (Figure 2F and Supplemental Figure 2D).

Older MplW514L mice exhibited obvious splenomegaly present in an allele dose–dependent manner (Figure 2G). Flow cytometric analysis showed that LSK (Lineage^–^Sca1^+^cKit^+^) population was significantly increased in the spleen of these MplW514L mice compared with their WT littermates, accompanied by marked expansion of megakaryocytes (CD41^+^), erythroid (TER119^+^), and myeloid (Gr1^+^Mac1^+^) lineages (Figure 2H and Supplemental Figure 2E). Spleen sections exhibited the disrupted splenic architecture with marked expansion of megakaryocytes in older heterozygous MplW514L-expressing mice, which was more severe in homozygous MplW514L-expressing mice (Figure 2I). These data indicated the extramedullary hematopoiesis in the older MplW514L mice.

Given the potential progression of ET to myelofibrosis, we performed reticulin staining and observed a marked increase in reticulin fibers in the BM and spleens of older MplW514L mice (Figure 2, C and I). A semiquantitative scoring system (scores from 0–3) revealed markedly higher fibrosis scores in MplW514L mice compared with heterozygous MplW514L-expressing ones and WT controls, indicating robust progression to myelofibrosis in the homozygous mutants (Figure 2J and Supplemental Table 3).

### MPN-initiating cells with MplW514L are enriched in the HSC-containing LSK compartment rather than in the GMP-containing LK compartment.

To determine if the MplW514L-driven MPN phenotype is transplantable, we transplanted total bone marrow cells from MplW514L mice or their WT littermates into lethally irradiated WT mice. The recipient mice with MplW514L-expressing BM exhibited the phenotypes in the primary MplW514L mice, including the elevated platelets (Supplemental Figure 3A), obvious splenomegaly accompanied by notable expansion of megakaryocytes and erythroid and myeloid cells (Supplemental Figure 3, B–D), and expansion of megakaryocytes in the bone marrow (Supplemental Figure 3E). Elevated platelets were also observed in the secondary transplant recipients (Supplemental Figure 3F). These results indicated that thrombocytosis and ET phenotypes in MplW514L mice are cell autonomous.

**Figure 3:**
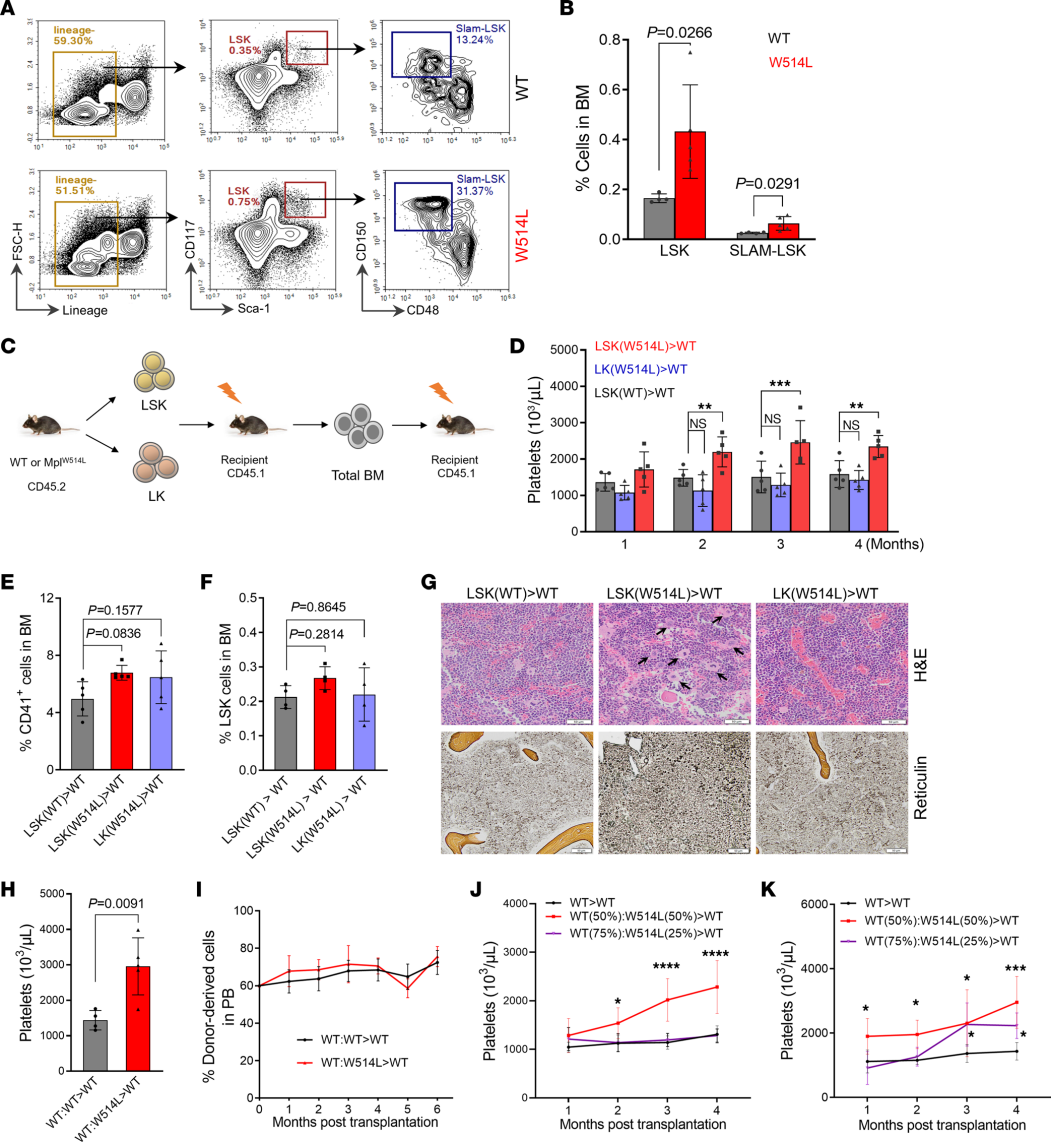
MplW514L-driven MPN-initiating cells are enriched in the HSC-containing LSK population. (**A**) Representative flow cytometric analysis of LSK and SLAM-LSK cells in the bone marrow of the indicated mice at 2 months of age. (**B**) Quantification of LSK and SLAM-LSK cells in **A**. *P* values were determined by 2-tailed unpaired Student’s *t* test. (**C**) Schematic of bone marrow transplantation using sorted LSK and LK cells from MplW514L mice or WT littermates. A total of 4 × 10^4^ LSKs or 4 × 10^5^ LKs were transplanted into lethally irradiated CD45.1 mice with 5 ×10^5^ supporting bone marrow cells. (**D**) Platelet parameters in peripheral blood of mice in **C**. *P* values were determined by 2-way ANOVA with Šidák’s multiple comparisons test. (**E–F**) Quantification of CD41^+^ and LSK cells in the bone marrow of mice in **C**. *P* values were determined by 1-way ANOVA with Dunnett’s multiple comparisons test. (**G**) Representative H&E staining and reticulin staining of bone marrow sections from the mice in **C**. Arrows indicated the megakaryocytic hyperplasia. Scale bar: 50 μm. (**H**) Platelet parameters after first competitive transplantation shown in Supplemental Figure 3H. *P* values were determined by 2-tailed unpaired Student’s *t* test. (**I**) Donor chimerism in peripheral blood after first competitive bone marrow transplantation shown in Supplemental Figure 3H. *n* = 5 per group. (**J–K**) Platelet parameters following transplantation with graded mutant HSCs from young (**J**, 2-month) or aged (**K**, 10-month) mice. *n* = 5–7 mice for each group. *P* values were determined by 2-way ANOVA with Šidák’s multiple comparisons test. All data were presented as mean ± SD. For dot plots, each dot representing one mouse. *P < 0.05, **P < 0.01, ***P < 0.001, ****P < 0.0001.

We assessed the hematopoietic stem cells in the bone marrow of MplW514L mice. Flow cytometric analysis revealed that LSK and SLAM-LSK (CD150^+^CD48^–^LSK) populations were significantly increased in MplW514L mice compared with their WT littermates (Figure 3, A and B), indicating that the MplW514L mutation has advanced effects on the LSK expansion.

To identify the hematopoietic developmental stage that contains the disease-initiating cells for MplW514L-driven MPN, we isolated and transplanted the LSK and LK (Lineage^–^Sca1^–^cKit^+^) populations from the bone marrow of MplW514L mice and their WT littermates into lethally irradiated recipient mice, respectively (Figure 3C). Recipients that received MplW514L-expressing LSK cells developed an ET-like MPN that was characterized by elevated platelet levels (Figure 3D), increased megakaryocytes and LSK cells in the bone marrow (Figure 3, E and F), and abnormal megakaryocytic hyperplasia (Figure 3G), phenocopying the ET in the primary mice. In contrast, recipients that received MplW514L-expressing LK cells failed to develop the ET-like MPN up to 4 months (Figure 3, D–G). We also performed secondary transplantation with unfractionated BM cells from the recipients of LSK cells, and found that the recipients still exhibited the elevated platelet levels (Supplemental Figure 3G). These findings demonstrated that MplW514L-expressing LSK cells but not committed progenitor cells are able to initiate and maintain the MPN in vivo.

To determine whether MplW514L confers an advantage to the LSK compartment, we performed a competitive bone marrow transplantation experiment. We transplanted a bone marrow mix from MplW514L mice and their WT littermates into lethally irradiated recipient mice (expressing CD45.1), in which the ratio of LSK cells from MplW514L mice and their WT littermates (expressing CD45.2) to WT mice (expressing CD45.2 and CD45.1) was 6:4 respectively (Supplemental Figure 3H). Recipients that received MplW514L-expressing BM developed an ET phenotype characterized by the elevated platelets in the peripheral blood (Figure 3H). We monitored these chimeric mice up to 6 months and found a constant percentage of CD45.2^+^ BM-derived cells in the peripheral blood between the groups (Figure 3I). The mice chimerized with MplW514L-expressing BM showed a mild expansion of megakaryocytes in the BM compared with the group that received WT BM (Supplemental Figure 3I). However, there was no obvious difference in the chimerism of LSK cells in the bone marrow between the groups at 24 weeks (Supplemental Figure 3J). Similar findings were also observed in the secondary BM transplanted mice (Supplemental Figure 3, K–M). These data demonstrated that MplW514L does not confer a competitive advantage to the HSCs.

Given that transplantable thrombocytosis and ET phenotypes were observed in transplants that received total MplW514L-expressing bone marrow but not in the competitive BMT setting, we next determined whether a specific dose of mutant HSC was required for disease initiation. We performed a titration of mutant HSCs in competitive transplantation assays that were composed of 50% and 25% mutant cells, respectively. Compared with recipients of young WT bone marrow, recipients of 50% young MplW514L-expressing bone marrow recapitulated the ET phenotype and exhibited significantly elevated platelet counts. In contrast, recipients of 25% young MplW514L-expressing bone marrow showed no increase in circulating platelets (Figure 3J). Interestingly, compared with recipients of aged WT bone marrow, recipients of both 50% and 25% aged MplW514L-expressing bone marrow developed the ET phenotype and displayed notable thrombocytosis (Figure 3K). We monitored these chimeric mice up to 4 months and observed a constant percentage of MplW514L-expressing BM-derived cells in the peripheral blood (Supplemental Figure 3, N and O). These findings suggest that disease initiation driven by MplW514L may depend on a quantitative threshold of mutant HSCs, and that aged MplW514L-expressing HSCs possess enhanced pathogenic potential.

### MplW514L enhanced megakaryocyte-lineage commitment within the mutant HSCs.

**Figure 4:**
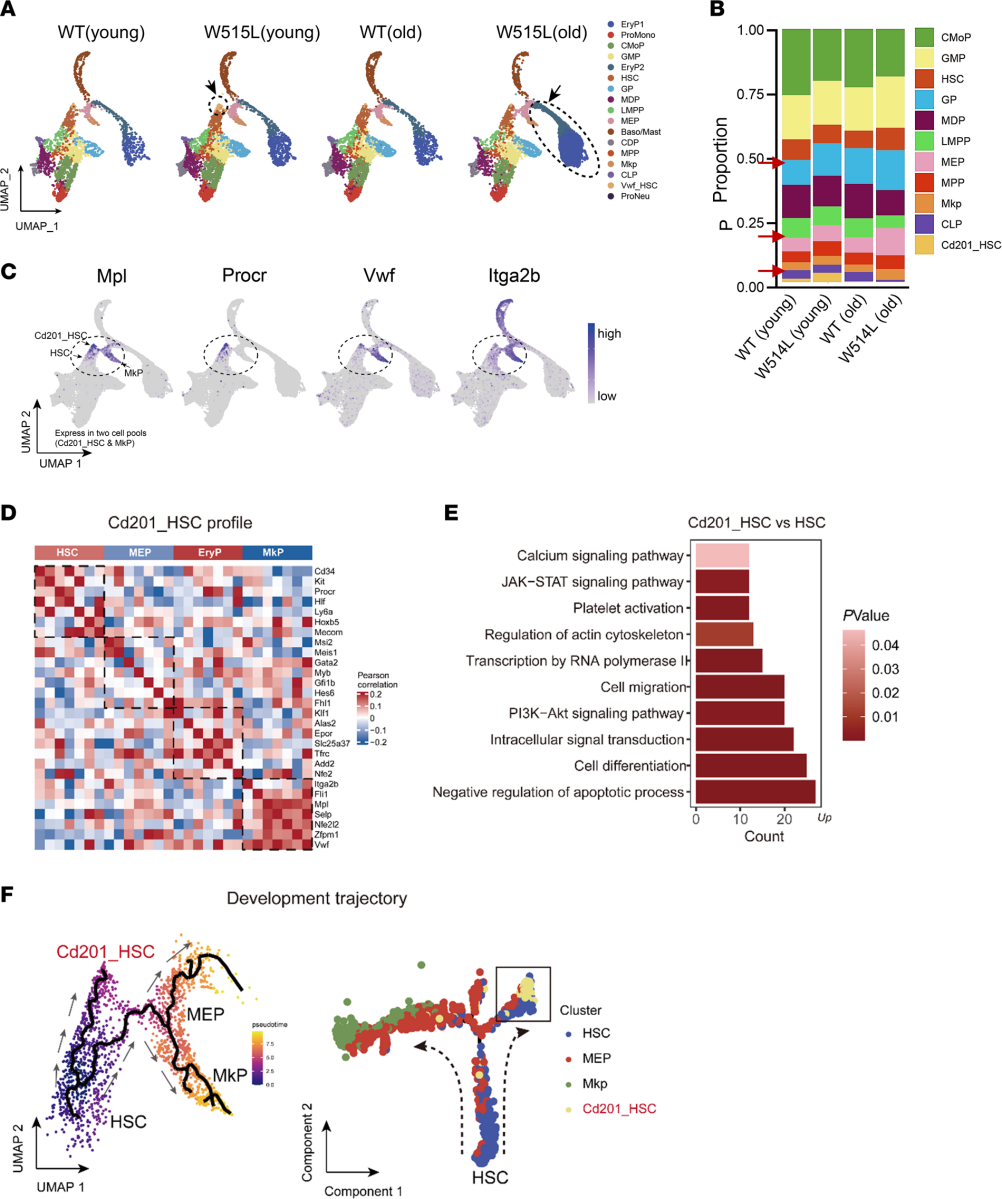
MplW514L mutation enhanced megakaryocyte lineage commitment in hematopoietic stem cells. (**A**) The UMAP visualization of all cells from the scRNA-seq datasets from young (2 months) and old (10 months) MplW514L mice and their WT littermates (*n* = 19,190 cells). (**B**) The stacked bar chart showed the proportions of each cluster in **A** excluding EryP1 and EryP2. Red arrows highlight the increased populations of young MplW514L mice compared with young WT control. (**C**) UMAP-based feature plots illustrated the expression levels of selected genes at single-cell resolution. (**D**) Gene pairwise Spearman correlation within the CD201_HSC. The heatmaps show increased expression of MkP-associated gene modules. (**E**) Enriched pathways of upregulated genes in CD201_HSCs compared with canonical HSC subpopulation. (**F**) Developmental trajectories of HSC, CD201_HSC, MEP, and MkP cells by Monocle (v2&v3). UMAP-based trajectory plot showing the differentiation progression of indicated cell populations, in which darker colors represent early differentiation stages and lighter colors indicate later stages (Left). Pseudotime-based dimension reduction plot, where arrows denote the inferred direction of cellular differentiation (Right).

To dissect the functional characterization of MplW514L in hematopoietic stem and progenitor cells, we performed single-cell RNA sequencing (scRNA-seq) in lineage-negative (Lin^–^) bone marrow cells from young (2 months) and aged (10 months) MplW514L mice and their WT littermates (Figure 4A). Following quality control, dimensionality reduction, and clustering, 17 distinct cell populations were identified, including HSC, CD201-high HSCs (CD201_HSC), multipotent progenitors (MPP), megakaryocyte-erythroid progenitors (MEP), megakaryocyte progenitors (MkP), erythroid progenitors (EryP1 and EryP2), lymphoid-primed multipotent progenitors (LMPP), granulocyte-monocyte progenitors (GMP), granulocyte progenitors (GP), neutrophil precursors (ProNeu), common monocyte progenitors (CMoPs), promonocytes (ProMono), monocyte-dendritic progenitors (MDP), common dendritic progenitors (CDP), common lymphoid progenitors (CLP), and basophil/mast cell progenitors (Baso/Mast) (Supplemental Figure 4, A and B). MplW514L mutation did not alter the global expression profile of *Mpl,* which was prominently expressed in HSCs and MkPs (Supplemental Figure 4C).

A novel cluster of CD201_HSC was identified, which were markedly expanded in young MplW514L mice but reduced in old MplW514L mice (Figure 4, A and B). This CD201_HSC subpopulation exhibited high expression of *CD201* (*Procr*) and *Mpl* (Figure 4C). Gene coexpression analysis showed that CD201^+^ HSCs display a transcriptional program resembling that of MkP-associated gene modules, indicating a priming toward megakaryocytic differentiation (Figure 4D). Compared with canonical HSCs, CD201_HSCs displayed increased transcriptional activity, a stress response signature, and a strong megakaryocyte-biased transcriptional profile (Figure 4E). Trajectory analysis also showed that CD201_HSCs originate from HSCs and diverge along a distinct lineage path separate from MEPs (Figure 4F). Furthermore, the transcriptional profile of CD201_HSCs closely resembles that of previously reported HSCs characterized by elevated levels of Vwf and Itga2b (CD41), which have been associated with a rapid megakaryocyte/platelet-generating potential (Figure 4C) (22, 23). Collectively, our results indicated that MplW514L mutation led to the expansion of a distinct CD201_HSC subpopulation, which conferred a megakaryocyte lineage bias within the HSCs.

**Figure 5:**
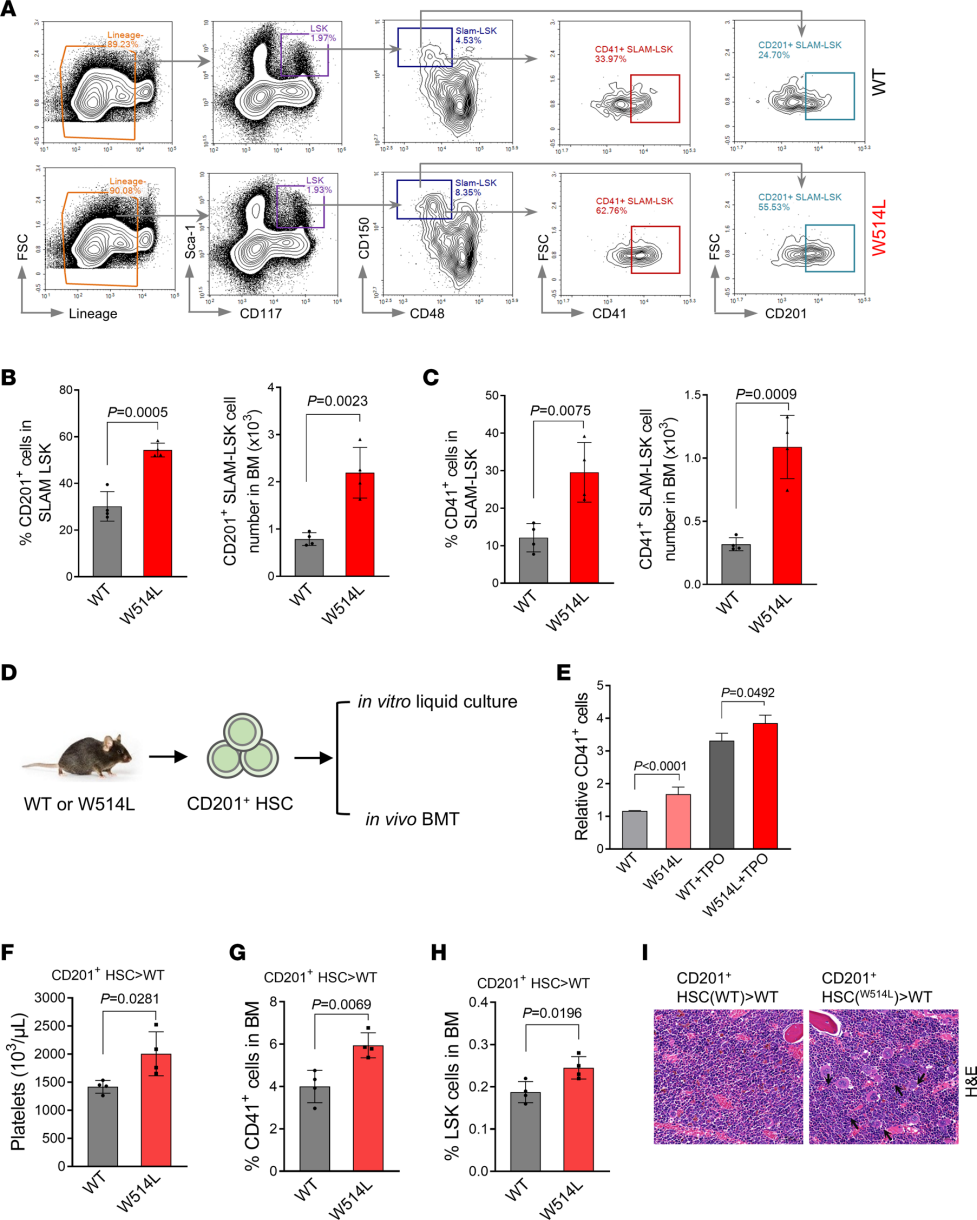
MplW514L mutation drove the expansion of CD201^+^ HSCs to initiate MPN. (**A**) Representative flow cytometric analysis of CD201^+^ SLAM-LSK and CD41^+^ SLAM-LSK cells in the bone marrow of the indicated mice at 2 months of age. (**B** and **C**) Quantification of the frequency and absolute cell number of CD201^+^ SLAM-LSK and CD41^+^ SLAM-LSK cells in **A**. (**D**) Schematic study of the biological functions of CD201^+^ HSCs. CD201^+^ HSCs were sorted from the bone marrow of MplW514L mice and their WT littermates and subjected to in vitro liquid culture and in vivo bone marrow transplantation. (**E**) Indicated CD201^+^ HSCs were cultured in TPO-containing medium for 7 days, and CD41^+^ cells count was detected by flow cytometry. Data were obtained from 3 different experiments. (**F**) Platelet parameters in peripheral blood of mice in **D**. A total of 2 × 10^3^ CD201^+^ HSCs were sorted from the bone marrow of MplW514L mice and their WT littermates and injected into lethally irradiated CD45.1 mice, together with 5 × 10^5^ bone marrow cells as supporting cells. The notation “donor cell population > recipient genotype” denotes the origin of donor cells and the recipient background. (**G** and **H**) Statistical analysis of CD41^+^ and LSK cells in the bone marrow of mice in **D**. (**I**) Representative H&E staining of bone marrow from the mice in **D**. Arrows indicated the megakaryocytic hyperplasia. Scale bar: 50 μm. All data are presented as mean ± SD. For dot plots, each dot represents one mouse. All *P* values were determined by 2-tailed unpaired Student’s *t* test.

To confirm the changes of this subpopulation, we performed the flow cytometric analysis in the bone marrow of young MplW514L mice (Figure 5A). We found that the frequency and cell count of CD201^+^ cells were significantly elevated in SLAM-LSK in the MplW514L mice compared with their WT littermates (Figure 5B). Similar expansion of CD41^+^ SLAM-LSK and Mpl^+^ SLAM-LSK was also observed in MplW514L mice, respectively (Figure 5C and Supplemental Figure 4D). To further explore the biological functions of CD201^+^ HSCs, we sorted CD201^+^ HSCs from the bone marrow of MplW514L mice and their WT littermates (Figure 5D). These cells were then subjected to in vitro liquid culture and in vivo bone marrow transplantation. Liquid culture analysis under megakaryocyte-promoting differentiation revealed that MplW514L-expressing CD201^+^ HSCs generated substantially more CD41^+^ cells than WT CD201^+^ HSCs (Figure 5E). Upon transplantation, recipients of MplW514L-expressing CD201^+^ HSCs developed an ET-like MPN, marked by elevated platelet counts (Figure 5F), increased megakaryocytes and LSK cells in the bone marrow (Figure 5, G and H), and abnormal megakaryocyte hyperplasia (Figure 5I). These findings demonstrated that MplW514L mutation drove the expansion of CD201^+^ HSC subpopulation with megakaryocyte lineage bias, which is sufficient to initiate MPN in vivo.

### MplW514L induced megakaryocytic skewing in the common myeloid progenitor compartment.

**Figure 6:**
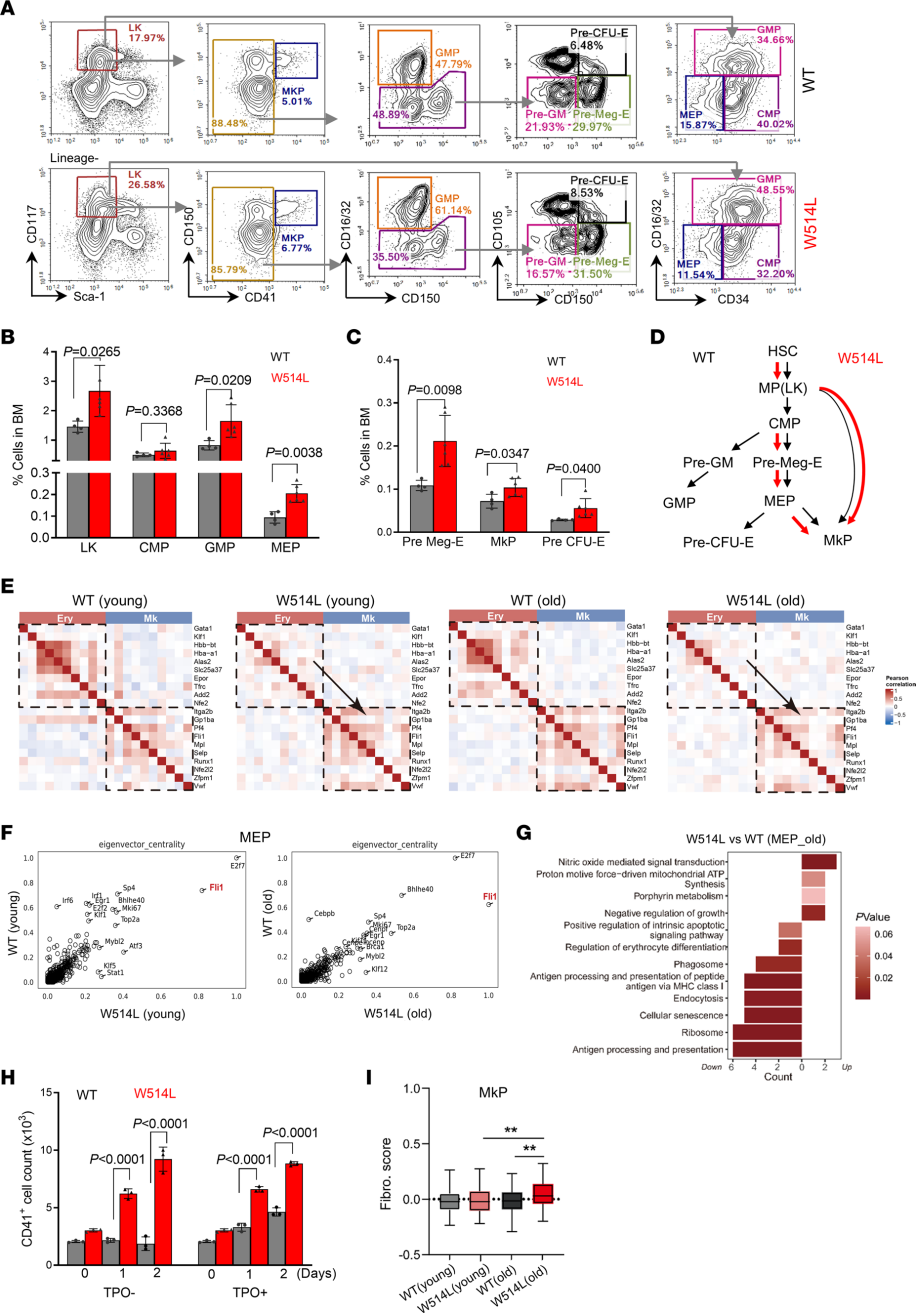
MplW514L mice exhibited megakaryocytic skewing in the myeloid progenitor compartment. (**A**) Representative flow cytometric analysis of LK, CMP, GMP, MEP, MKP, Pre Meg-E, and Pre CFU-E in bone marrow of the indicated mice at 10 months. (**B** and **C**) Quantification of the proportions of indicated cell population in **A**. Data are presented as mean ± SD, with each dot representing one mouse. Data shown were representative of 2 independent experiments. *P* values were determined by 2-tailed unpaired Student’s *t* test. (**D**) Schematic summarizing effects of MplW514L mutation on hematopoietic progenitor compartment as in **A**. Black arrows represent normal differentiation, while red arrows indicated the enhanced megakaryocyte-biased lineage commitment. (**E**) Pairwise Spearman correlation of genes in MEPs from scRNA-seq data (Figure 4A), showing bias toward MkP-associated modules in MplW514L mice. (**F**) CellOracle-based evaluation of eigenvector centrality in MEP cells in **E**. Regulatory network analysis was performed using CellOracle to assess eigenvector centrality scores of genes in MEP cells across 4 groups, indicating the relative influence of individual genes within the inferred gene regulatory networks. (**G**) Pathway enrichment analysis of differentially expressed genes in MEP cells in **E**. (**H**) Statistical analysis of CD41^+^ cells from the cultured bone marrow cells from MplW514L mice and their WT littermates. Lineage-negative cells were isolated and cultured in the medium with or without thrombopoietin (TPO) for 3 days. Data were obtained from 3 independent experiments and presented as mean ± SD. *P* values were determined by 2-way ANOVA with Šidák’s multiple comparisons test. (**I**) Fibrosis-promoting evaluation of the indicated megakaryocyte progenitor (MkP) from the scRNA-seq datasets in Figure 4A, using the AddModuleScore function in Seurat. All gene sets are described in Supplemental Table 4. *P* values were determined by 1-way ANOVA with Tukey’s multiple comparisons test. **P < 0.01.

scRNA-seq analysis also showed a marked alteration in hematopoietic progenitor cells from MplW514L mice, including an increase in MEPs and GPs, and excessive expansion of erythroid progenitors (EryP) in old MplW514L mice (Figure 4, A and B). We then performed flowcytometric analysis to dissect the lineage compartment of hematopoietic progenitor cells in MplW514L mice. We found that immunophenotypically defined myeloid progenitor cells (Lin^–^Sca^–^cKit^+^, LK) were increased in MplW514L mice compared with their WT littermates (Figure 6, A and B). The elevated MPs are mainly due to the notable expansion of MEP (Lin^–^Sca^–^cKit^+^CD16/32^–^CD34^–^) and GMP (Lin^–^Sca^–^cKit^+^CD16/32^–^CD34^+^), but not common myeloid progenitors (Lin^–^Sca^–^cKit^+^CD16/32^–^CD34^+^, CMP) (Figure 6, A and B). According to the expression of CD105, CD150, and CD41(17), subsequent analyses of MPs revealed that the frequency of premegakaryocyte erythroid progenitor cells (Lin^–^Sca^–^cKit^+^CD41^–^CD16/32^–^CD150^+^CD105^–^, Pre Meg-E) was also increased in MplW514L mice. Notably, MkPs (Lin^–^Sca^–^cKit^+^CD150^+^CD41^+^), which are restricted to megakaryocytic fate, were significantly expanded in MplW514L mice compared with their WT littermates (Figure 6C). These data indicate that MplW514L mutation enhanced the megakaryocytic skewing of hematopoietic progenitor cells, increasing megakaryocyte-erythroid progenitors over granulocyte-monocyte progenitors (Figure 6D).

Single-cell transcriptomic analysis revealed a megakaryocyte lineage bias in MEP of MplW514L mice (Figure 6E). In line with this altered profile, *Fli1*, a transcription factor critical for megakaryocyte differentiation (24), showed high expression in MplW514L-expressing MEP, as indicated by CellOracle analysis (Figure 6F). In the MEPs from old MplW514L mice, multiple signaling pathways related to erythroid differentiation were significantly downregulated, including ribosome synthesis, endocytosis, and direct regulation of erythrocyte differentiation (Figure 6G).

Although preerythrocyte colony-forming units (Lin^–^Sca^–^cKit^+^CD41^–^CD16/32^–^CD150^+^CD105^+^, Pre CFU-E cells) were significantly expanded in MplW514L mice compared with their WT littermates (Figure 6C), transcriptomic analysis of erythroid progenitors revealed impaired features of erythroid progenitors in aged MplW514L mice (Supplemental Figure 5A). Furthermore, enrichment analysis of differentially expressed genes and transcription factor activity indicated a developmental blockade within the erythroid progenitors in these mice (Supplemental Figure 5, B and C). These findings may account for the excess expansion of erythroid precursors but developing anemia in aged MplW514L mice. To further evaluate erythroid differentiation, we cultured sorted Pre CFU-E cells from MplW514L mice and their WT controls in erythropoietin-containing medium. After 1 day of culture, the erythroid progenitors and precursors were markedly lower in the MplW514L group compared with that of WT control group (Supplemental Figure 5, D–F).

It has been demonstrated that MplW514L mutation leads to Mpl signaling activation that is hypersensitive to its ligand TPO-binding even in the absence of TPO (25). We cultured lineage-negative BM cells derived from MplW514L mice and their WT controls in media containing TPO. As we expected, WT BM cells exhibited no proliferation without TPO but obvious expansion upon the stimulation of TPO. Strikingly, MplW514L-expressing BM cells showed a marked megakaryocyte-expansion that is independent of TPO stimulation (Figure 6H). Furthermore, in line with the age-dependent myelofibrosis in MplW514L mice, we found that a profibrotic score in MkPs was markedly elevated in aged MplW514L mice but not young ones (Figure 6I).

### Fedratinib alleviated MPN features in MplW514L mice but failed to eliminate disease-initiating cells.

We demonstrated that MplW514L-driven MPN-initiating cells were particularly enriched in the HSC population, in which MplW514L mutation led to the expansion of CD201_HSC subpopulation, which conferred a megakaryocyte lineage bias. To evaluate the effect of JAK2 inhibitors that are in clinical use for the treatment of MPNs, we first examined the JAK2 signaling in MplW514L HSC. Although JAK-STAT signaling was markedly enriched in CD201_HSC compared with the classical HSC, MplW514L mutation did not confer a higher JAK-STAT signaling score in CD201_HSC than in WT CD201_HSC (Supplemental Figure 6, A and B). This was further confirmed by the comparable STAT5 phosphorylation in CD201^+^ HSCs from MplW514L mice and their WT controls (Supplemental Figure 6C). These findings indicated that a JAK/STAT-independent mechanism may drive the expansion of disease-initiating HSC in this model.

**Figure 7:**
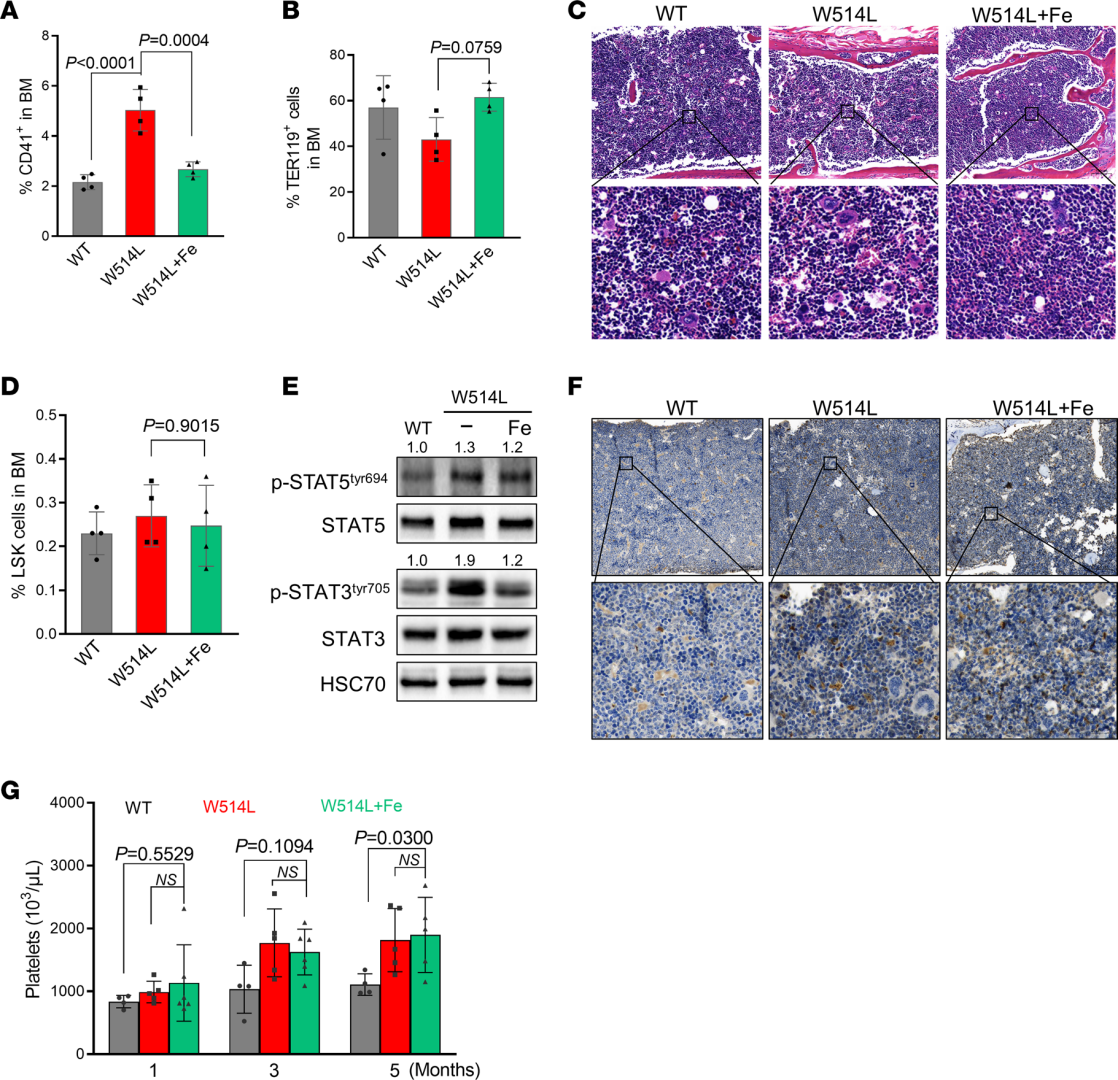
Fedratinib alleviated MPN features in MplW514L mice but failed to eliminate disease-initiating cells. (A-B) Quantification of CD41^+^ and TER119^+^ cell proportions in the bone marrow of MplW514L mice and their WT littermates after 4-weeks of fedratinib treatment. *P* values were determined by 1-way ANOVA with Tukey’s multiple comparisons test. (**C**) Representative H&E staining of bone marrow from the mice in **A**. Scale bar: 50 μm. (**D**) Statistical analysis of Lineage^–^Sca^+^cKit^+^ (LSK) cell proportions in the bone marrow from the mice in **A**. *P* values were determined by 1-way ANOVA with Tukey’s multiple comparisons test. (**E**) Immunoblotting analysis of indicated proteins in bone marrow mononuclear cells from Fedratinib-treated MplW514L mice, with HSC70 as a loading control. (**F**) Representative images of CD201 immunohistochemical staining in bone marrow sections from vehicle- and fedratinib-treated MplW514L mice. Scale bar: 50 μm. (**G**) Platelet parameters in peripheral blood of secondary recipient mice that received the unfractionated bone marrow cells from indicated mice in **A**. Total bone marrow were transplanted into lethally irradiated WT mice. *P* values were determined by 2-way ANOVA with Šidák’s multiple comparisons test. All data were presented as mean ± SD, with each dot representing one mouse.

To directly evaluate the therapeutic effect of JAK2 inhibitors on MplW514L mice, Fedratinib, a JAK2 inhibitor was administered twice daily for 4 weeks (60 mg/kg, oral gavage). Fedratinib treatment had no effects on the elevated platelets in the MplW514L mice (Supplemental Figure 6D), which may be due to the long half life of platelets and the limited treatment duration. Notably, flow cytometric analysis revealed that elevated CD41^+^ cells were significantly reduced after the treatment of Fe, accompanied with marked recovery of diminished TER119^+^ cells (Figure 7, A and B). Similar findings were also observed in the histopathological analysis of bone marrow (Figure 7C). These data indicated that Fedratinib treatment led to the reduction of megakaryocytic hyperplasia in bone marrow.

However, no obvious differences in LSK frequencies were observed in the bone marrow of Fedratinib-treated mice compared with vehicle controls (Figure 7D). Bone marrow cells were collected from treated MplW514L mice and subjected to Western blotting analysis. Compared with WT littermates, bone marrow cells from MplW514L mice exhibited a constitutive increase of phosphorylation of STAT5 and STAT3, which was markedly reduced in the Fedratinib-treated mice (Figure 7E). These data indicate Fedratinib treatment efficiently inhibits JAK-STAT signaling.

Our single-cell RNA-seq analysis had shown that CD201 is highly and specifically expressed in HSCs, establishing it as a robust marker for in situ identification of HSCs in bone marrow sections. We then performed immunohistochemistry (IHC) staining for CD201 to assess HSCs in the bone marrow sections from these mice. IHC analysis of CD201 revealed that the frequency of CD201^+^ cells in the bone marrow remained unchanged following Fedratinib treatment, indicating that JAK2 inhibitors did not affect mutant HSCs (Figure 7F). This is consistent with the notion that JAK inhibition mitigates disease manifestations but does not eradicate the HSC-like population. To further investigate the functional consequence of this persistence, we transplanted bone marrow from Fedratinib-treated MplW514L mice into lethally irradiated WT mice. Both groups of mice that received BM treated with or without Fedratinib exhibited the continuously elevated platelets up to 5 months after transplantation, suggesting that Fedratinib had no effect on the disease-initiating cells in MplW514L mice (Figure 7G).

## Discussion

We described an MplW514L (equivalent human MPLW515L) knock-in model to compare the effects of physiological MplW514L expression on the MPN phenotype. A single *MplW514L* allele is sufficient to develop an MPN resembling human ET, which includes excess thrombocytosis, megakaryocytic hyperplasia, and abnormal megakaryocyte morphology in the bone marrow. However, homozygous MplW514L expression not only resulted in an ET-like phenotype associated with markedly greater thrombocytosis and megakaryocytic hyperplasia but also led to the accelerated progression to myelofibrosis compared with heterozygous MplW514L-expressing mice. These findings mirror the clinical observations that heterozygous MPL mutations are found in patients with ET and PMF, while homozygous MPL mutations are only detected in patients with PMF (6).

We demonstrated that disease-initiating cells are particularly enriched in the HSC-containing LSK population in MplW514L mice. Our scRNA-seq data showed that *Mpl* is uniquely highly expressed in HSC and MkPs, which is not disrupted by the MplW514L mutation. Given that TPO/MPL (TPOR) signaling is the primary driver of megakaryocyte differentiation and platelet production (4), MplW514L mutation leading to spontaneous activation of MPL enhances the megakaryocytic skewing of hematopoietic stem and multipotent progenitor cells that account for the ET and megakaryocytic hyperplasia. In addition, aged MplW514L mice exhibited a high risk of progression to myelofibrosis, in which the burden of immature megakaryocytes in the bone marrow is continuously driven by MplW514L. In line with this, increased megakaryocytes in the bone marrow had been associated with marrow fibrosis formation (26, 27).

We identified a novel cluster of CD201^+^ HSC within the HSC compartment, whose transcriptional profile closely resembles that of previously reported HSC subpopulation marked by elevated Vwf and CD41 expression and megakaryocyte/platelet-generating potential (23, 28). CD201 is widely recognized as a marker of quiescent HSCs and plays a crucial role in maintaining stem cell homeostasis (29, 30). Our data demonstrated that MplW514L mutation drove the expansion of CD201^+^ HSC subpopulation with megakaryocyte lineage bias, which is sufficient to initiate MPN in vivo. Indeed, a direct differentiation route from HSCs to MkPs has recently been shown to enhance TPO signaling, with high CD201 expression marking the “leading edge” of lineage differentiation (31). TPO is one of 3 key cytokines essential for HSC maintenance and expansion (32). TPO treatment also drives a bias for megakaryopoiesis and platelet production without causing obvious HSC expansion in mice (33, 34). Therefore, the disease phenotype induced by the MplW514L mutation arises from the pathological expansion of CD201^+^ “leading edge” HSCs, which specifically accelerate the aforementioned “short route” to platelet production.

Our data demonstrate that MplW514L mutation led to a substantial increase in HSCs but did not confer competitive advantage to HSCs in vivo. Furthermore, disease initiation driven by MplW514L may depend on a quantitative threshold of mutant HSCs, and aged MplW514L mutant HSCs possess enhanced pathogenic potential. The elevated CD20^+^ HSCs subpopulation in young MplW514L mice was significantly reduced in aged MplW514L mice, indicating that these megakaryocyte-committed HSCs have low self-renewal/maintenance activity and undergo the exhaustion. Similar to our observations in mice, the chimerism of MplW514L-positive CD34^+^ cells from patients with PMF gradually decreased in NOD/SCID mice and was absent in secondary transplant recipients (12). It is possible that MplW514L mutation leads to the hyperactivation of MPL signaling that, in turn, enhances the typical HSC expansion. However, this advantage is offset by the increased but exhausted megakaryocyte-committed HSCs in the HSC pool.

Although we cannot rule out the possibility that treatment with a higher dose or prolonged administration of Fedratinib might diminish the MPN-initiating population, our observations indicate that Fedratinib treatment reduced MplW514L-expressing hematopoietic progenitors without altering MplW514L-expressing HSCs, particularly the CD201^+^ HSC population. Indeed, JAK/STAT signaling was not substantially upregulated in MplW514L-expressing CD201^+^ HSC compared with WT CD201^+^ HSC, suggesting that expansion of the MPN-initiating population in MplW514L mice is driven by a mechanism distinct from canonical JAK activation. Several potential mechanisms may account for the resistance of MplW514L mutant HSCs to JAK inhibition. First, compensatory activation of alternative pathways, such as MAPK/ERK and PI3K/AKT, may sustain HSC survival independently of JAK/STAT signaling (35). Second, persistent inflammatory signaling may provide a protective microenvironment that supports mutant HSC maintenance (36, 37). Third, metabolic reprogramming, particularly enhanced glycolytic activity, may contribute to stem cell persistence and therapeutic resistance, consistent with emerging evidence linking metabolism to MPN progression (38, 39). Finally, the intrinsic quiescence of HSCs may limit the efficacy of JAK inhibitors, which primarily target actively proliferating cells (40, 41).

Our findings suggest that targeting JAK/STAT-independent pathways downstream of Mpl, or disrupting metabolic and inflammatory dependencies, may represent a key strategy for eradicating MPN-initiating stem cells. Recent studies have identified multiple signaling pathways involved in MPN pathogenesis, including epigenetic regulation (such as TET2 and ASXL1) and RNA splicing (such as SRSF2 and SF3B1) (42, 43). Notably, TET2 deletion has been shown to confer a competitive advantage to stem cells through epigenetic upregulation of Mpl expression and enhancement of TPO receptor signaling (44). Thus, aberrant activation of the TPO signaling pathway emerges as a critical convergence point driving abnormal hematopoiesis. Targeting TPO signaling or blocking downstream noncanonical pathways provide promising new directions for the treatment of MPN driven by Mpl mutations and TPO signaling abnormalities.

In summary, we reported an MplW514L knock-in mouse model that develops an MPN resembling human ET. We demonstrated that the MplW514L-expressing HSC compartment had the unique disease-initiating capacity but did not exhibit a competitive advantage over WT HSCs. We found that MplW514L expression led to a marked expansion of megakaryocyte-biased stem cells within the HSC pool, which were skewed toward the megakaryocytic lineage. JAK2 inhibitor treatment alleviated the MPN phenotype but failed to eliminate the disease-initiating population. These findings underscore the consequences of physiological expression of MPLW515L mutation on HSCs and also provide a valuable model to evaluate the therapies for MPLW515L-positive MPN.

## Methods

### Sex as a biological variable.

Our study examined male and female animals, and similar findings are reported for both sexes. The MplW514L knock-in mice were generated via CRISPR/Cas9-mediated recombination (see Supplemental Information for full details). Vav-Cre mice were purchased from the Jackson Laboratory.

### Flow cytometric analysis.

Single-cell suspensions of bone marrow, spleen, and peripheral blood were prepared and stained as previously described (45). Detailed information about the antibodies is provided in Supplemental Table 1. For LSK and LK cell sorting, lineage-negative (Lin^–^) cells were isolated using a commercial lineage depletion kit (BD, #559971) according to the manufacturer’s instructions. After staining the cells with the indicated antibodies, they were sorted using a FACS sorter (Beckman Coulter).

### Bone marrow transplantation.

Noncompetitive and competitive BM transplantation were performed as previously described (46).

### Geneset scoring.

The gene set scores of each cell were estimated using the AddModuleScore function in Seurat, which calculated the average expression levels of each program (cluster) on single cell level, subtracted by the aggregated expression of control feature sets. All analyzed features are binned based on averaged expression, and the control features are randomly selected from each bin. The pro-fibrotic gene set and the regulation of receptor signaling pathway via JAK-STAT gene set are described in Supplemental Tables 4 and 5.

### CellOracle analysis.

CellOracle (version 0.18.0) was applied to single-cell RNA-seq data to reconstruct gene regulatory networks and evaluate transcription factor activity. Default parameters were used unless otherwise specified: GRN inference was performed with a 10-nearest neighbor graph (KN *N* = 10), and eigenvector centrality scores were calculated to identify key regulators.

### Cell development analysis.

The macrophage development locus was analyzed by Monocle2 (version 2.26.0) and Monocle3 (version 1.4.25) (balanced for equal cell numbers). Monocle utilizes the strategy of ordering single cells in pseudotime, placing them along a trajectory corresponding to a biological process such as cell differentiation by taking advantage of individual cell’s asynchronous progression of those processes.

### Statistics.

Statistical analysis was performed using Prism 8 (GraphPad Software). Data are presented as mean ± SD unless otherwise indicated. Differences between 2 groups were analyzed using a 2-tailed unpaired Student’s *t* test. For comparisons among multiple groups, 1-way ANOVA followed by Dunnett’s multiple comparisons test (for comparisons against a single control) or Tukey’s multiple comparisons test (for all pairwise comparisons) was used. For experiments involving 2 independent variables, 2-way ANOVA followed by Šidák’s multiple comparisons test was performed. The *P* value of less than 0.05 was considered statistically significant.

### Study approval.

All animal studies were performed in accordance with the Guidelines for the Care and Use of Laboratory Animals and were approved by the Institutional Animal Care and Use Committees at Shandong University (#20021).

### Data availability.

ScRNA-Seq data in this study have been deposited in the China National Center for Bioinformation/Beijing Institute of Genomics database at https://ngdc.cncb.ac.cn (GSA: CRA029479). All data values are available in supplemental materials or the Supporting data values document.

## Author contributions

BZ designed and guided research; SZ, JL, LW, YW and MX performed the experiments; SZ, JL, Yuan Li, Yanxia Li, GD, SW, ZC and BZ analyzed the data; BZ and SZ wrote the original draft; SZ, Yuan Li, Yanmei Li, ZC and BZ reviewed and edited the manuscript. All authors have read and agreed to the published version of the manuscript. The order of co–first authors was determined by the relative level of contribution, with the first author listed having made the greater overall contribution.

## Conflict of interest

The authors have declared that no conflict of interest exists.

## Funding support

- National Key Research and Development Program of China (2024YFC2510500, to BZ).
- National Natural Science Foundation of China (81874294, BZ).
- Natural Science Foundation of Shandong Province (ZR2024MH065, to YL).
- National Natural Science Foundation of China (82371789 and 82170173, to ZC).
- The Key Program of Innovation Improvement of Small and Medium-sized Enterprises of Shandong Province in China (2023TSGC0717, to BZ).

## Supplementary Material

Supplemental data

Unedited blot and gel images

Supplemental tables 1-5

Supporting data values

## Version 1 04/23/2026

In-Press Preview

## Version 2 06/01/2026

Electronic publication
