# Supplementary material for: Physiological MplW514L expression in hematopoietic stem cell causes an essential thrombocythemia and progressive myelofibrosis
Source: J Clin Invest. 2026 Apr 23;136(11):e199690. doi: 10.1172/JCI199690 (PMC13221233; doi:10.1172/JCI199690)
Supplement: Supplemental data [file jci-136-199690-s083.pdf]

Physiological MplW514L expression in hematopoietic stem cell causes an essential thrombocythemia and progressive myelofibrosis

Shujing Zhang[1,2,3,4#], Jingjing Liu[5#], Yuan Li[1,2,3,4], Yi Wang[1,2,3,4], Lingling Wang[1,2,3,4], Miaomiao Xu[1,2,3,4], Yanxia Li[1,2,3,4], Ge Dong[5], Shanshan Wang[5], Yanmei Li[6], Zhigang Cai[5*], Baobing Zhao[1,2,3*]

[1]State Key Laboratory of Discovery and Utilization of Functional Components in Traditional Chinese Medicine, Cheeloo College of Medicine, Shandong University, Jinan, Shandong, China;

[2]Key Laboratory of Chemical Biology (Ministry of Education), School of Pharmaceutical Sciences, Cheeloo College of Medicine, Shandong University, Jinan, China；

[3]NMPA Key Laboratory for Technology Research and Evaluation of Drug Products, School of Pharmaceutical Sciences, Cheeloo College of Medicine, Shandong University, Jinan, Shandong, China;

[4]Department of Pharmacology, School of Pharmaceutical Sciences, Cheeloo College of Medicine, Shandong University, Jinan, China；

[5]State Key Laboratory of Experimental Hematology, Tianjin Key Laboratory of Inflammatory Biology, Department of Pharmacology, School of Basic Medical Science, Tianjin Medical University, Tianjin, China.

[6]The Key Laboratory of Chemistry for Natural Products of Guizhou Province and Chinese Academic of Sciences, Guiyang, China.

[#]These authors contributed equally to this work.

[*]Correspondence to:
Baobing Zhao, Ph.D., baobingzh@sdu.edu.cn;
Zhigang Cai, Ph.D., us36zcai@tmu.edu.cn.

Supplementary Materials and Methods

Supplementary Figures and Figure Legends

Supplementary Tables

Supplementary Materials and Methods

Generation of conditional MplW514L Knock-in Mice

To create a mouse MPL conditional knock-in model in C57BL/6J mice, CRISPR/Cas-mediated genome engineering was used. In brief, gRNAs targeting the mouse MPL gene (gRNA1: 5'-CAATTAGTGCAAGGCACCGAGGG-3' and gRNA2: 5'-TGCGTTTGGAGACGCTCGCCAGG-3') were co-injected with a donor vector containing the "loxP-endogenous SA of intron 8-CDS of exon 9-12-3'UTR-3*SV40 pA-loxP" cassette and Cas9 mRNA into fertilized mouse eggs to generate targeted conditional knock-in offspring. Conditional knock-in offspring were identified by PCR, followed by sequence analysis. Standard techniques were used to create chimeric mice. Floxed MplW514L littermate mice (FL/FL) were genotyped by PCR using primers MplW514L-FL (F: 5'-CTGCTAAAGTGGCAATTTCCTGC-3' and R: 5'-CAATGTATCTTATCATGTCTGGATCCCC-3'). Excision after Vav-Cre recombination was confirmed by PCR with primers to detect a portion remaining post-excision (MplW514L KI-F: 5'-TCACTGTGCTCCCTCGGTGCATA-3' and MplW514L KI-R: 5'-CACGCAGCGTAGACCAGCTTCAG-3').

Western blotting analyses

Cells were lysed in RIPA buffer (Beyotime, P0013B) with protease and phosphatase inhibitor mixture (Beyotime, P1045) for 30 min, then centrifuged at 12000 rpm, 4 °C. The protein expression levels were determined by staining with primary antibodies. The antibodies used in this study were listed in Supplemental Table S1.

mRNA extraction and Quantitative RT-PCR

Total RNA from CRC cells was extracted using the Trizol reagent (Invitrogen, 15596026CN) according to the manufacturer's instructions. RNA quality and

quantity were determined using a Nano Drop and Agilent 2100 bioanalyzer (Thermo). RNA was reversely transcribed into cDNA using RT reagent kit (TaKaRa, RR047). A SYBR Green PCR kit (TaKaRa, RR420) was used for quantitative real-time PCR and results were quantified with an Applied Biosystem System (ABI) with appropriate primers. The human housekeeping gene GAPDH was used as the RNA-loading control. Gene expression was determined by the delta CT method ($2^{-\Delta\Delta Ct}$). ΔCt = (Ct target gene – Ct housekeeping). The primers used for the PCR were Mpl-forward, TCCGAGACTGCTTGGATCAC; Mpl -reverse, GTGTAGGTCTGGAAGCGAGG; GAPDH-forward, AGGTCGGTGTGAACGGATTTG; and GAPDH-reverse, TGTAGACCATGTAGTTGAGGTCA.

Frequency of Vav-Cre recombination

The frequency of Vav-Cre recombination in MplW514L mice were determined as previously described(1). Briefly, plasmids encoding MplW514L and Mpl were mixed at various ratios and used as templates for PCR amplification with Mpl primers, which amplify both Mpl and MplW514L alleles. The PCR products were sequenced, and a standard curve was generated by plotting the actual T/G mutation ratio (X-axis) against the quantified peak height ratio (Y-axis). For experimental samples, cDNA derived from bone marrow cells of $Mpl^{+/+}$, $Mpl^{W514L/+}$, and $Mpl^{W514L/W514L}$ mice was amplified using Mpl primers, and the PCR products were directly sequenced. In the sequencing chromatograms, the T peak corresponds to the MplW514L mutant allele, while the G peak represents the Mpl allele. Peak heights of T and G were quantified using PeakFit software. The percentage of each allele was calculated as follows:

%T = (T peak height) / (T peak height + G peak height) × 100%

%G = (G peak height) / (T peak height + G peak height) × 100%

Bone marrow cells *in vitro* culture

lineage-negative (Lin^-) cells were isolated using a commercial lineage depletion

kit (BD, 559971) according to the manufacturer's instructions. For the erythroid differentiation, Lin^- cells were cultured in Iscove's Modified Dulbecco's Medium (Gibco) containing 15% fetal bovine serum (FBS, Biological Industries), 1% Bovine Serum Albumin (StemCell), 10 ug/ml human Insulin (Sigma-I9278), 200 µg/ml human holo-transferrin (Sigma-T0665), 10^{-4} M β-mecaptoethanol, and 2 U/ml human Epo (Amgen). After 24 h of culture, flow cytometric analysis was performed based on the surface expression of TER119 and CD71.

For the megakaryocytic differentiation, Lin^- cells were cultured in RPMI Media 1640 supplemented with 10% FBS and 20 ng/mL thrombopoietin, followed by the flow cytometric analysis based on the surface expression of CD41.

For expansion in liquid culture, the sorted $CD201^+$ HSC and $CD201^-$ HSC cells (500 cells/100ul in a 96 well plate) were grown in IMDM supplemented with 10% FBS, L-glutamine (2 mM), and the following cytokines: SCF (50 ng/mL), TPO (100ng/ mL), IL-3 (10 ng/mL) and IL-6 (10 ng/mL). After 7 days of cultivation in the incubator, CD41 antibody staining was performed and flow cytometry analysis was performed.

For intracellular staining of phospho-STAT5 (p-STAT5), mouse bone marrow $CD201^+$ HSC cells were stimulated with TPO (50 ng/mL) at 37 °C for 10 minutes. Following the manufacturer's protocol, the cells were fixed with Fixation/Permeabilization working solution (BD), followed by staining with anti-phospho-Stat5.

Blood and tissue analysis

Peripheral blood cell count in mice was performed using a fully automated animal blood cell analyzer (Mindray BC-2800 Vet). Mouse serum thrombopoietin (TPO) levels were determined by a mouse TPO ELISA kit (Elabscience, E-EL-M0640) according to the manufacturer's instructions.

For histopathological analysis, mouse bone and spleen samples were fixed in

10% neutral-buffered formalin and embedded in paraffin. Tissue sections were stained with hematoxylin, eosin, and reticulin stain. Evaluation of disease phenotypes (ET-like and MF-like features) was performed as described below.

Assessment of ET-like phenotype (thrombocytosis)

To determine the penetrance of the ET-like phenotype, thrombocytosis was defined as a platelet count exceeding the mean value for age-matched wild-type controls at each time point. The number of mice per genotype (N) and the proportion of animals exceeding this predefined threshold are summarized in Supplemental Table S2. All animals were included in the analysis, with no exclusions.

Assessment of Bone Marrow Fibrosis (MF-like Phenotype)

The assessment of bone marrow fibrosis-like phenotype was based on the following four independent criteria, established in accordance with the World Health Organization (WHO) diagnostic framework for bone marrow proliferative tumors(2). Animals that met at least two of these criteria were classified as exhibiting a phenotype similar to MF. The number, age distribution, and proportion of mice meeting these criteria for each genotype were detailed in Supplemental Table S3.

(1) Characteristic Atypical Megakaryocyte Clusters: Defined on H&E-stained sections as ≥3 megakaryocytes in direct contact, displaying nuclear hyper-chromasia, irregular folding, and/or other atypical features. A sample was considered positive if such clusters were observed in ≥3 non-overlapping fields of view(3).

(2) Fibrosis Grading: Silver staining was performed, and fibrosis was semi-quantitatively scored according to the European consensus grading system (MF-0 to MF-3)(4).

(3) Splenomegaly: Spleen weight was measured during dissection. Splenomegaly was defined as a spleen weight exceeding the average weight

of age-matched wild-type control mice.

(4) Decreased Blood Cells: Based on peripheral blood analysis, anemia was defined as a red blood cell count or hematocrit below the mean of wild-type controls; leukopenia was defined as a white blood cell count lower than the mean of wild-type controls(5).

Isolation of single cells for Single-cell RNA-seq

Bone marrow was harvested from the indicated mice. Lineage-negative (Lin^-) cells were isolated using a commercial lineage depletion kit (BD, #559971) according to the manufacturer's instructions. Cell debris and dead cells (Miltenyi, #130-109-398/130-090-101) were removed to obtain the final sample. The scRNA-seq datasets were deposited in the GSA under accession code CRA029479.

Library construction and sequencing of mouse Single-cell RNA-seq

SeekOne Digital Droplet Single Cell 3' library preparation kit to construct Single cell RNA sequencing (scRNA-seq) library: 1) About 12000 single cells were mixed with reverse transcription reagents and added to the sample hole of SeekOne chip, and then barcode hydrogel beads (BHBs) and partitioning oil were added to the corresponding hole of the chip respectively; 2) After milk droplet formation, reverse transcription and inactivation were performed; 3) cDNA was purified from broken milk drops and amplified by PCR; 4) The amplified cDNA product was cleaned, segmented, end-repaired, added a tail and connected to the sequencing connector; 5) The DNA of gene 3' polyA was amplified by indexed PCR, including cell Barcode and Unique Molecular Indentifier (UMI). 6) Amplified cDNA product was then cleaned up using the SPRIselect Reagent Kit. Illumina NovaSeq 6000 was used for second-generation sequencing, and the reading length was PE150.

scRNA-seq data processing

The reads of each gene in the scRNA-seq sample of mouse were quantified against the mm10 mouse reference genome using the SeekSoulTools (version 1.2.0). Seurat objects were generated using Seurat 4.4.0, and the quality control criteria were that the number of cell gene expression was greater than 200 and less than 7500, and the proportion of mitochondrial genes was less than 20%. After filtering, 19190 cells and 23865 genes were obtained. Next, NormalizeData was applied for normalization; FindVariableFeatures was used to find high-variable genes, ScaleData was converted to Z-score for PCA dimensionality reduction; RunHarmony was used to remove batch effect; FindNeighbors and FindClusters function were used for cell cluster analysis; RunUMAP was nonlinear dimensionality reduction and visualization.

To integrate data sets from different batches into a shared space for unsupervised clustering, we use the harmony algorithm for batch effect correction. The highly expressed genes in each cluster were identified according to FindAllMarkers, and the top genes were regarded as marker genes. We annotated cell types for all clusters based on known marker genes.

Pathway enrichment

The differential gene between the disease group and the control group was calculated by Findmarkers with the parameter "min.pct"=0.25 and "logfc.threshold"=0.25. Differentially expressed genes were subjected to pathway enrichment analysis using the DAVID online tool (Database for Annotation, Visualization and Integrated Discovery). Functional annotation was performed using Gene Ontology biological processes and KEGG pathways, with a cutoff of $P<0.05$ for significance.

JAK2 Inhibitor Studies

Fedratinib hydrochloride hydrate was Purchased from TargetMol (#T9251). For in vivo use, stock solutions were diluted in sterile water.

Supplementary Figures and Figure Legends

Figure S1 MplW514L knock-in mice exhibited thrombocytosis, related to Figure 1.

(**A**) Alignment analysis of the human and mouse MPL gene and protein sequences with the mutation site. (**B**) The chromatogram of sequencing PCR products of mixed plasmids encoding Mpl and MplW514L with indicated ratio. (**C**) Standard curve generated by the true T (peak identified mutant)/G (peak identified WT allele) ratio (X-axis) and the measured peak height ratio (Y-axis) established in B. (**D**) Statistical analysis of thrombopoietin (TPO) levels in the serum of the indicated mice. Data were presented as mean ± SD, with each dot representing one mouse. *P* values were determined by one-way ANOVA with Dunnett's multiple comparisons test. (**E-F**) Monocyte and neutrophil parameters in the peripheral blood of indicated mice at 2-12 months of age. Data were presented as mean ± SD, with each dot representing one mouse. *P* values were determined by two-way ANOVA with Sidak's multiple comparisons test. $^{*}P < 0.05$, $^{**}P < 0.01$, $^{***}P < 0.001$, *NS* represent no significant difference.

Figure S2 MplW514L knock-in mice recapitulated an ET-like MPN and progressed myelofibrosis, related to Figure 2.

(**A**) Total bone marrow cell counts of the indicated mice at 2 months of age. Data were presented as mean ± SD, with each dot representing one mouse. (**B**) Representative flow cytometric analysis and quantification of $CD41^{+}$ cells in the bone marrow of the indicated mice at 2 months of age. Data were presented as mean ± SD, with each dot representing one mouse. (**C**) Representative hematoxylin and eosin (H&E) staining of bone marrow from the mice in A. Arrows indicated the megakaryocytic hyperplasia. Scale bar: 50 μm. (**D**) Representative flow cytometric analysis of $CD41^{+}$, $TER119^{+}$, and $Mac1^{+}Gr1^{+}$ cells in the bone marrow of the indicated mice at 10 months of age. (**E**) Representative flow cytometric analysis of $Lineage^{-}Sca^{+}cKit^{+}$ cell (LSK), $CD41^{+}$,

TER119$^+$ and Mac1$^+$Gr1$^+$ cells in the spleen of the indicated mice at 10 months of age. All *P* values were determined by two-way ANOVA with Sidak's multiple comparisons test.

Figure S3. MplW514L-driven MPN-initiating cells are particularly enriched in the HSC-containing LSK population, related to Figure 3.

(**A**) Platelet parameters in the peripheral blood of recipient mice transplanted with Mpl^{W514L} or WT bone marrow at 3-24 weeks. *P* values were determined by two-tailed unpaired Student's *t* test. (**B**) Schematic of spleen and spleen weight statistics in recipient mice as in A after 6-months of bone marrow transplantation. *P* values were determined by two-tailed unpaired Student's *t* test. (**C-E**) Quantification of CD41$^+$ (**C**), Mac1$^+$Gr1$^+$ and TER119$^+$ (**D**) cells in the spleen, and CD41$^+$ cells in the bone marrow (**E**) of recipient mice as in **A**. *P* values were determined by two-tailed unpaired Student's *t* test. (**F**) Platelet parameters after transplantation with total bone marrow cells from 6-month BMT mice in A. *P* values were determined by two-way ANOVA with Sidak's multiple comparisons test. (**G**) Platelet parameters in secondary recipients receiving unfractionated BM cells from LSK-transplanted mice. *P* values were determined by two-way ANOVA with Sidak's multiple comparisons test. (**H**) Schematic of serial competitive bone marrow transplantation with the indicated mice bone marrow. (**I-J**) Donor-derived CD41$^+$ and LSK cells in bone marrow after 6-months of the first competitive BMT. *P* values were determined by two-tailed unpaired Student's *t* test. (**K**) Donor chimerism in peripheral blood after secondary transplantation. Data were obtained from 5 mice in each group. (**L-M**) Donor-derived CD41$^+$ (**L**) and LSK (**M**) cells after secondary transplantation as shown in Fig.S3H. *P* values were determined by two-tailed unpaired Student's *t* test. (**N-O**) Donor chimerism after transplantation with graded mutant HSCs from young (**N**) or aged (**O**) mice. Data were obtained from 5 mice in each group. All data were presented as mean ± SD. For dot plots, each dot representing one mouse.

Figure S4. MplW514L mutation enhanced megakaryocyte lineage commitment in hematopoietic stem cells, related to Figure 4.

(**A**) Uniform manifold approximation and projection (UMAP) visualization of lineage-negative HSPCs from the scRNA-seq datasets from young (2 months) and old (10 months) MplW514L mice and their WT littermates (n=19190 cells). (**B**) Marker genes for each identified cluster in A. (**C**) Expression profiles of the indicated genes in each cluster cells across the four samples as in A. (**D**) Quantification of Mpl^{+} SLAM-LSK cell frequency in the bone marrow of mice as in Fig.4G. Data were presented as mean ± SD, with each dot representing one mouse. *P* values were determined by two-tailed unpaired Student's *t* test.

Figure S5. MplW514L mice exhibited megakaryocytic skewing in the myeloid progenitor compartment, related to Figure 6.

(**A**) Gene pairwise Spearman correlation within the 2EryPs (EryP1 + EryP2) from the scRNA-seq datasets in Fig.4A. The heatmaps showed that the old MplW514L group exhibits a transcriptional bias toward MkP-associated gene modules. (**B**) Pathway enrichment analysis of differentially expressed genes in 2EryPs cells from old MplW514L mice compared to old WT mice. (**C**) CellOracle-based evaluation of eigenvector centrality in 2EryPs cells as in B. (**D**) Representative flow cytometric analysis of erythroid differentiation in sorted Pre CFU-E cells from the indicated mice after 1-day culture with erythropoietin. (**E-F**) Quantification of the frequency and cell count of indicated subpopulations in D. Data were obtained from three independent experiments and presented as mean ± SD. *P* values were determined by two-tailed unpaired Student's *t* test.

Figure S6. Fedratinib had no effects on the disease-initiating cells in MplW514L mice, related to Figure 7.

(A) Violin plots showing JAK-STAT signaling scores in HSC cluster versus CD201_HSC cluster from the scRNA-seq datasets in Fig.4A. (**B**) JAK-STAT

signaling scores of CD201_HSC from young MplW514L mice and their WT littermates with the scRNA-seq datasets in Fig.4A. (**C**) Representative flow cytometric analysis of phospho-STAT5 (p-STAT5) levels in CD201^{+} HSC cells of WT and MplW514L mice after starvation or stimulation with Thrombopoietin (TPO, 50 ng/mL) for 10 minutes. Representative data were obtained from one of three independent experiments. (**D**) Platelet parameters in peripheral blood of indicated mice with 4-weeks of fedratinib treatment. Data were presented as mean ± SD, with each dot representing one mouse.

Supplementary Tables

Table S1 Antibodies and commercial reagents used in this study

Table S2 The penetrance of thrombocytosis in MplW514L mice, including detailed genotype- and age-specific distributions

Table S3 The penetrance of myelofibrosis in MplW514L mice, including detailed genotype- and age-specific distributions

Table S4 Pro-fibrotic gene set

Table S5 Regulation of receptor signaling pathway via JAK-STAT gene set

References

1. Akada H, et al. Conditional expression of heterozygous or homozygous Jak2V617F from its endogenous promoter induces a polycythemia vera-like disease. *Blood.* 2010;115(17):3589-97.
2. Arber DA, et al. The 2016 revision to the World Health Organization classification of myeloid neoplasms and acute leukemia. *Blood.* 2016;127(20):2391-405.
3. Thiele J, et al. Bone marrow histopathology in myeloproliferative disorders--current diagnostic approach. *Semin Hematol.* 2005;42(4):184-95.

4. Thiele J, et al. European consensus on grading bone marrow fibrosis and assessment of cellularity. *Haematologica.* 2005;90(8):1128-32.
5. Barbui T, et al. The 2016 WHO classification and diagnostic criteria for myeloproliferative neoplasms: document summary and in-depth discussion. *Blood Cancer J.* 2018;8(2):15.

Fig.S1

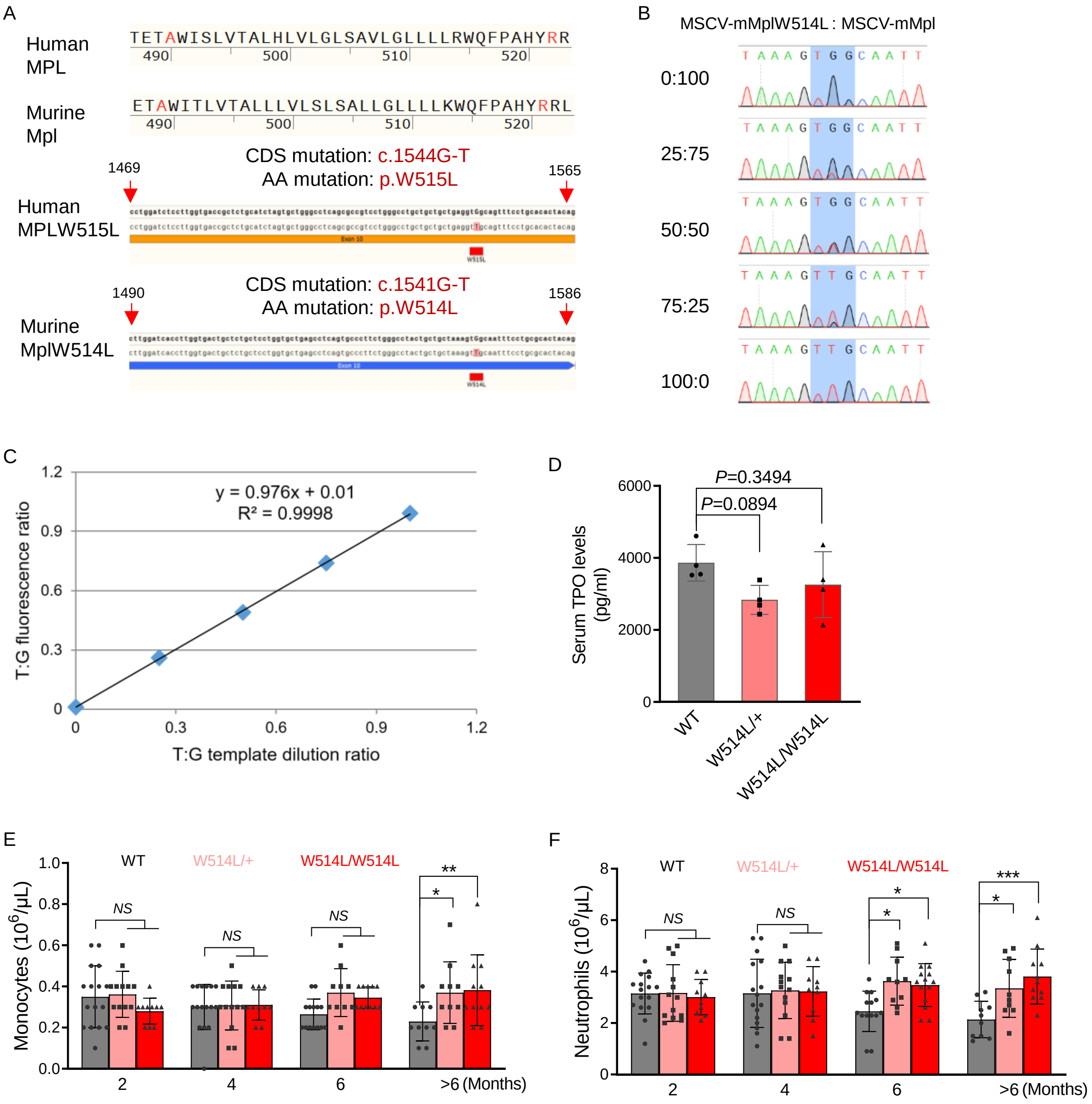

A
Human MPL
TETAWISLVTALHLVLGLSAVLGLLLLRWQFPAHYRR
490 500 510 520
Murine Mpl
ETAWITLVTALLLVLSLSALLGLLLLKWQFPAHYRRL
490 500 510 520
CDS mutation: c.1544G-T
AA mutation: p.W515L
1469
1565
Human MPLW515L
CDS mutation: c.1541G-T
AA mutation: p.W514L
1490
1586
Murine MplW514L
B
MSCV-mMplW514L : MSCV-mMpl
0:100
25:75
50:50
75:25
100:0
C
y = 0.976x + 0.01
R² = 0.9998
T:G fluorescence ratio
T:G template dilution ratio
D
P=0.3494
P=0.0894
Serum TPO levels (pg/ml)
WT
W514L/+
W514L/W514L
E
WT
W514L/+
W514L/W514L
Monocytes (10^6/μL)
NS
**
*
2
4
6
>6 (Months)
F
Neutrophils (10^6/μL)
***
*
2
4
6
>6 (Months)

Fig.S2

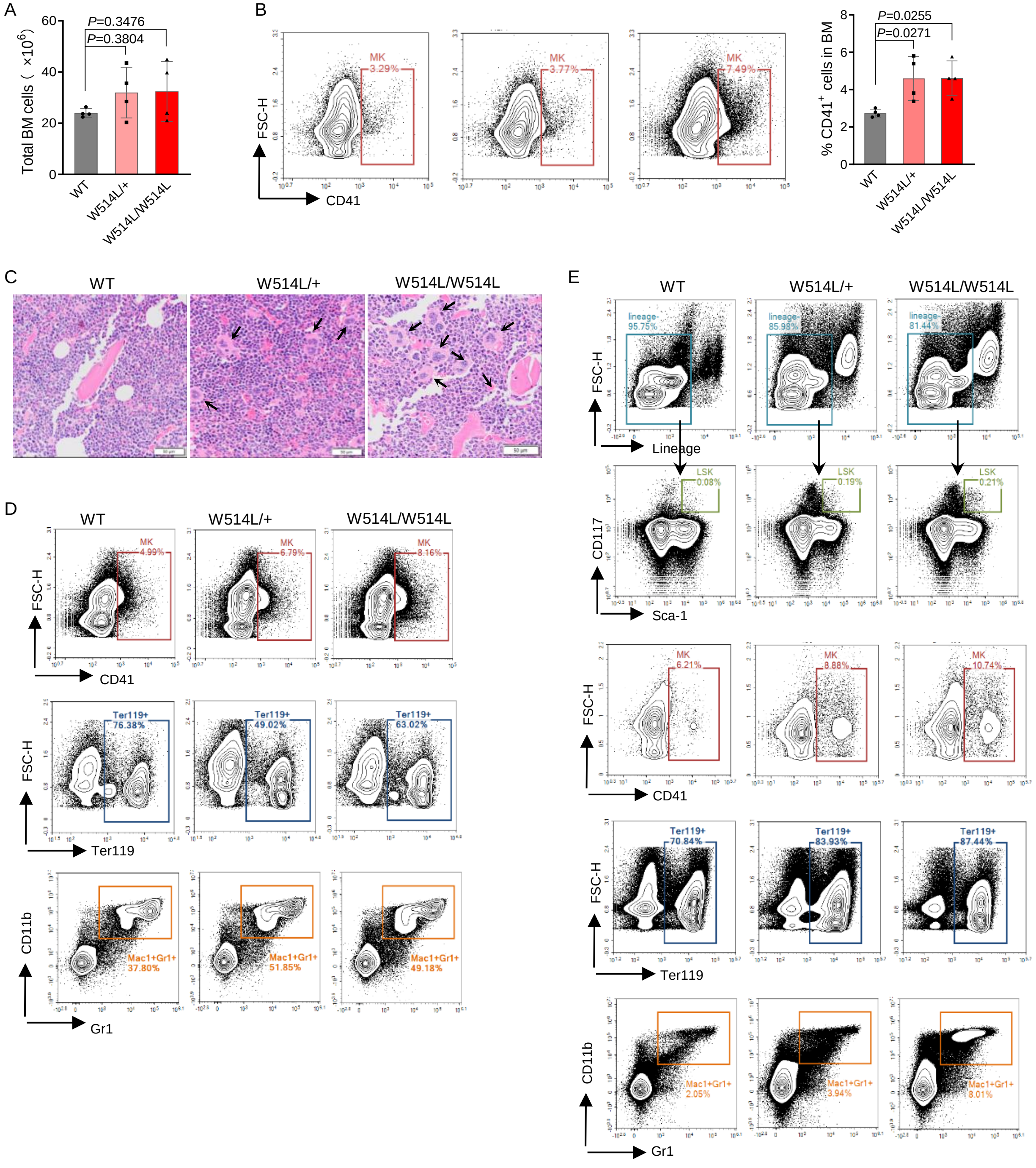

A
Total BM cells（×10^6）
P=0.3476
P=0.3804
WT
W514L/+
W514L/W514L
B
FSC-H
CD41
MK 3.29%
MK 3.77%
MK 7.49%
% CD41+ cells in BM
P=0.0255
P=0.0271
C
WT
W514L/+
W514L/W514L
D
MK 4.99%
MK 6.79%
MK 8.16%
Ter119
Ter119+ 76.38%
Ter119+ 49.02%
Ter119+ 63.02%
CD11b
Gr1
Mac1+Gr1+ 37.80%
Mac1+Gr1+ 51.85%
Mac1+Gr1+ 49.18%
E
Lineage
lineage- 95.75%
lineage- 85.98%
lineage- 81.44%
CD117
Sca-1
LSK 0.08%
LSK 0.19%
LSK 0.21%
MK 6.21%
MK 8.88%
MK 10.74%
Ter119+ 70.84%
Ter119+ 83.93%
Ter119+ 87.44%
Mac1+Gr1+ 2.05%
Mac1+Gr1+ 3.94%
Mac1+Gr1+ 8.01%

Fig.S3

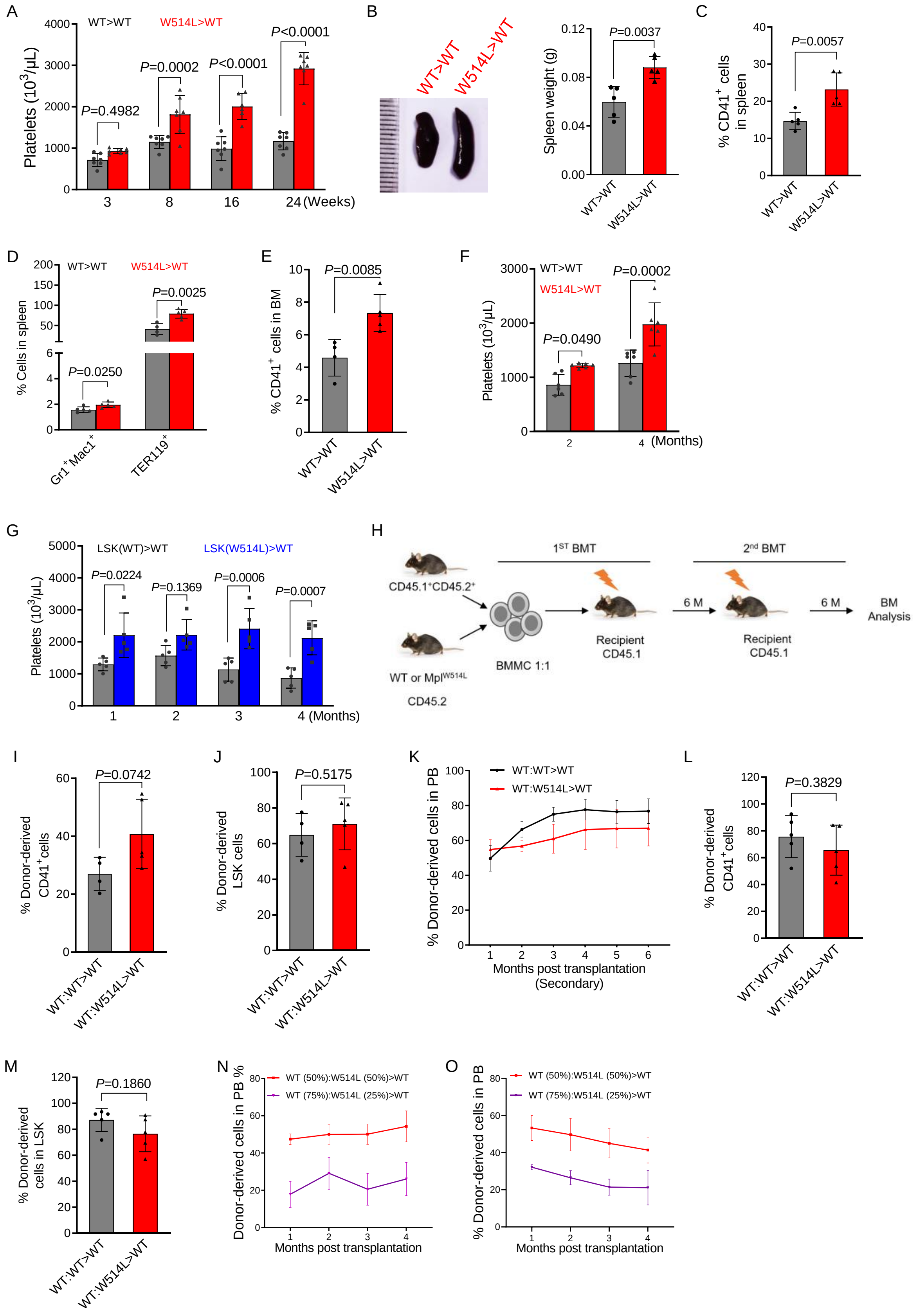
A
WT>WT
W514L>WT
Platelets (10³/μL)
P=0.4982
P=0.0002
P<0.0001
P<0.0001
3
8
16
24 (Weeks)
B
WT>WT
W514L>WT
Spleen weight (g)
P=0.0037
C
% CD41+ cells in spleen
P=0.0057
D
% Cells in spleen
P=0.0025
P=0.0250
Gr1+Mac1+
TER119+
E
% CD41+ cells in BM
P=0.0085
F
P=0.0490
P=0.0002
2
4 (Months)
G
LSK(WT)>WT
LSK(W514L)>WT
P=0.0224
P=0.1369
P=0.0006
P=0.0007
1
2
3
4 (Months)
H
1ST BMT
2nd BMT
CD45.1+CD45.2+
WT or MplW514L
CD45.2
BMMC 1:1
Recipient
CD45.1
6 M
BM Analysis
I
% Donor-derived CD41+cells
P=0.0742
WT:WT>WT
WT:W514L>WT
J
% Donor-derived LSK cells
P=0.5175
K
% Donor-derived cells in PB
WT:WT>WT
WT:W514L>WT
Months post transplantation
(Secondary)
L
% Donor-derived CD41+cells
P=0.3829
M
% Donor-derived cells in LSK
P=0.1860
N
Donor-derived cells in PB %
WT (50%):W514L (50%)>WT
WT (75%):W514L (25%)>WT
Months post transplantation
O
% Donor-derived cells in PB

Fig.S4

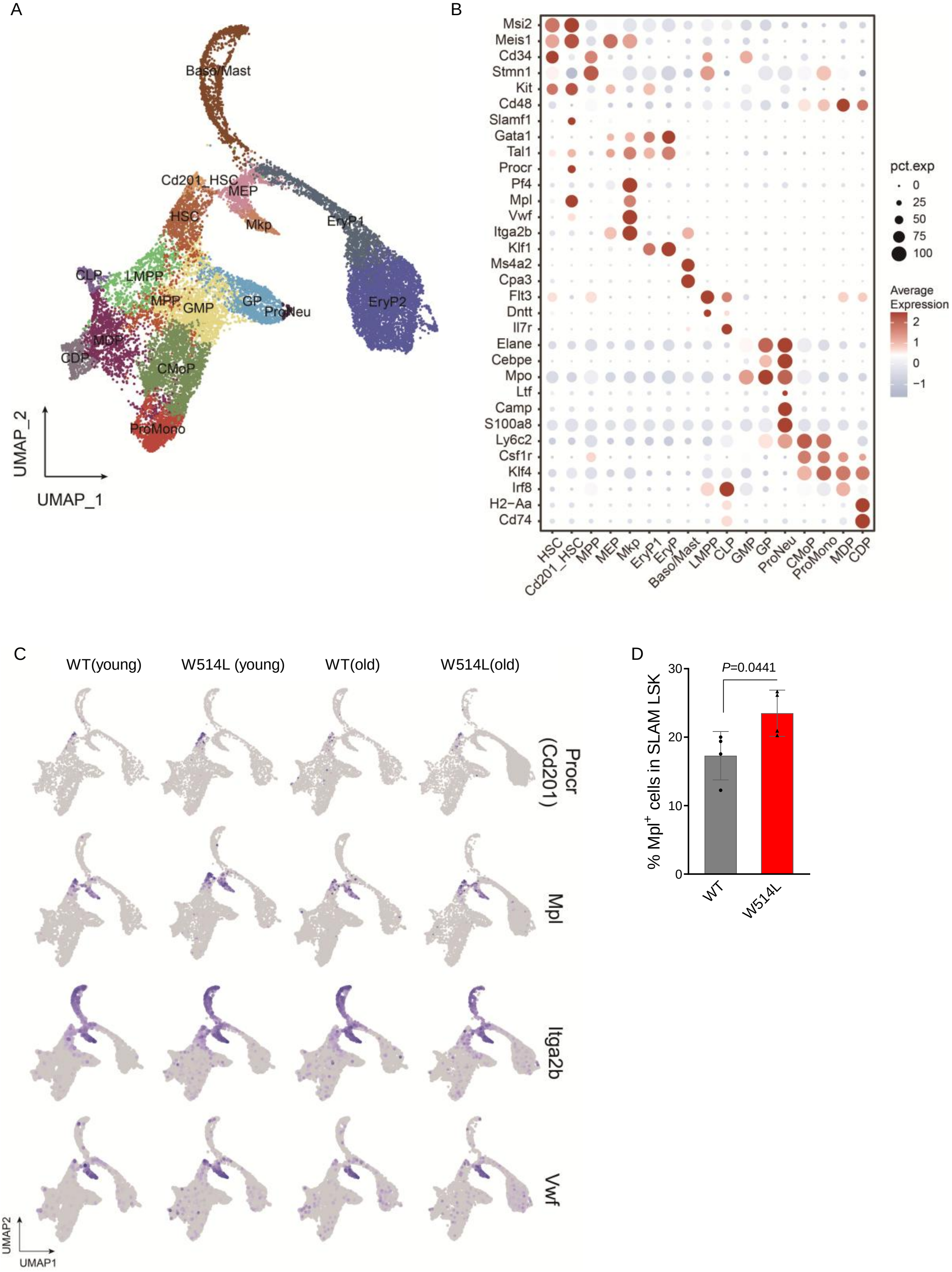
A
Baso/Mast
Cd201_HSC
MEP
HSC
Mkp
EryP1
CLP
LMPP
MPP
GMP
GP
ProNeu
EryP2
MDP
CDP
CMoP
ProMono
UMAP_2
UMAP_1
B
Msi2
Meis1
Cd34
Stmn1
Kit
Cd48
Slamf1
Gata1
Tal1
Procr
Pf4
Mpl
Vwf
Itga2b
Klf1
Ms4a2
Cpa3
Flt3
Dntt
Il7r
Elane
Cebpe
Mpo
Ltf
Camp
S100a8
Ly6c2
Csf1r
Klf4
Irf8
H2−Aa
Cd74
HSC
Cd201_HSC
MPP
MEP
Mkp
EryP1
EryP
Baso/Mast
LMPP
CLP
GMP
GP
ProNeu
CMoP
ProMono
MDP
CDP
pct.exp
0
25
50
75
100
Average Expression
2
1
0
−1
C
WT(young)
W514L (young)
WT(old)
W514L(old)
Procr (Cd201)
Mpl
Itga2b
Vwf
UMAP2
UMAP1
D
P=0.0441
% Mpl+ cells in SLAM LSK
30
20
10
0
WT
W514L

Fig.S5

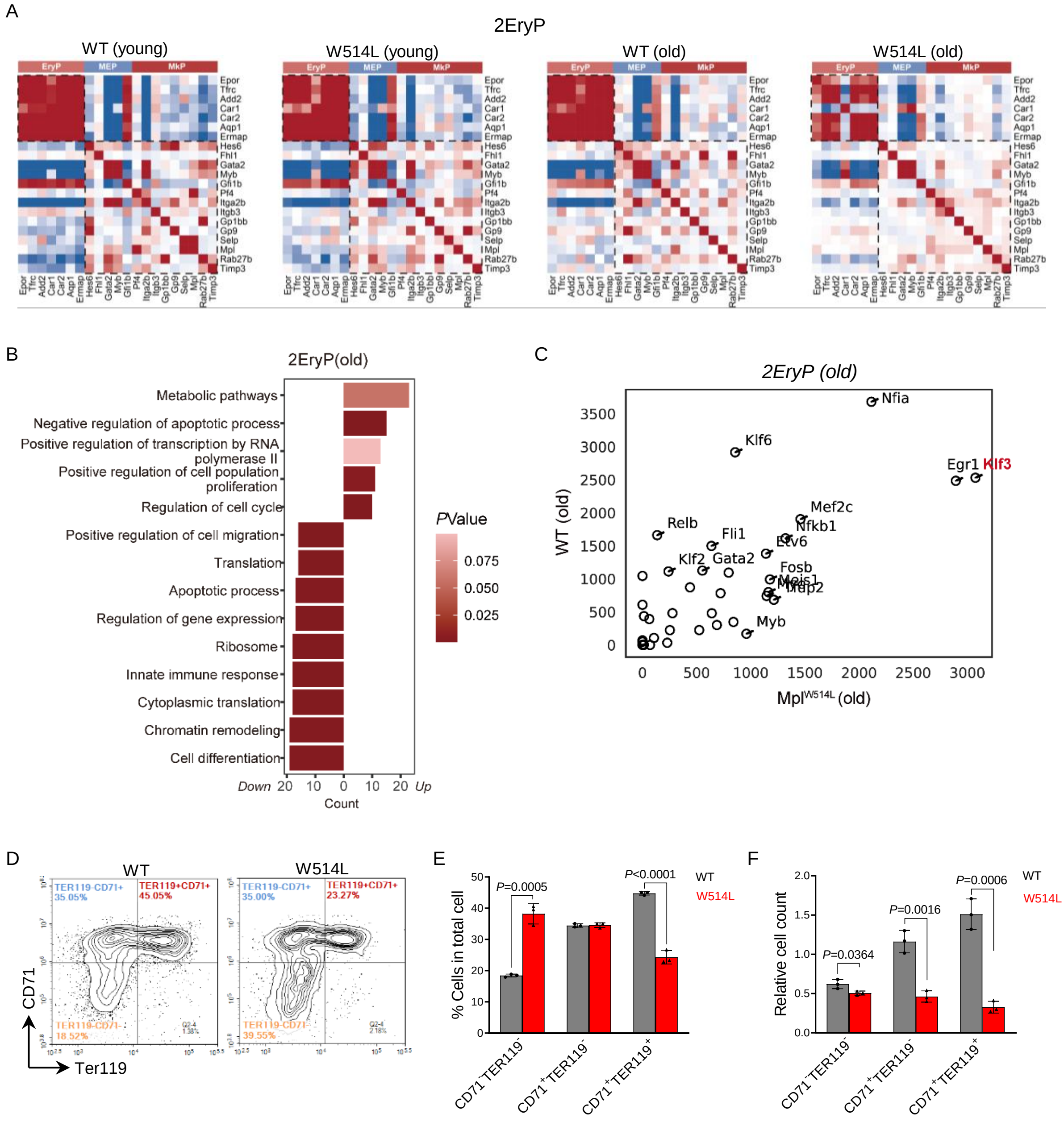
A
2EryP
WT (young)
W514L (young)
WT (old)
W514L (old)
EryP
MEP
MkP
Epor
Tfrc
Add2
Car1
Car2
Aqp1
Ermap
Hes6
Fhl1
Gata2
Myb
Gfi1b
Pf4
Itga2b
Itgb3
Gp1bb
Gp9
Selp
Mpl
Rab27b
Timp3
B
2EryP(old)
Metabolic pathways
Negative regulation of apoptotic process
Positive regulation of transcription by RNA polymerase II
Positive regulation of cell population proliferation
Regulation of cell cycle
Positive regulation of cell migration
Translation
Apoptotic process
Regulation of gene expression
Ribosome
Innate immune response
Cytoplasmic translation
Chromatin remodeling
Cell differentiation
Down 20 10 0 10 20 Up
Count
PValue
0.075
0.050
0.025
C
2EryP (old)
Nfia
Klf6
Egr1 Klf3
Mef2c
Nfkb1
Relb
Fli1
Etv6
Klf2
Gata2
Fosb
Meis1
Myb
WT (old)
Mpl^W514L (old)
3500
3000
2500
2000
1500
1000
500
0
0 500 1000 1500 2000 2500 3000
D
WT
W514L
TER119-CD71+
35.05%
TER119+CD71+
45.05%
TER119-CD71-
18.52%
TER119-CD71+
35.00%
TER119+CD71+
23.27%
TER119-CD71-
39.55%
CD71
Ter119
E
% Cells in total cell
P=0.0005
P<0.0001
WT
W514L
CD71-TER119-
CD71+TER119-
CD71+TER119+
F
Relative cell count
P=0.0364
P=0.0016
P=0.0006
WT
W514L
CD71-TER119-
CD71+TER119-
CD71+TER119+

Fig.S5

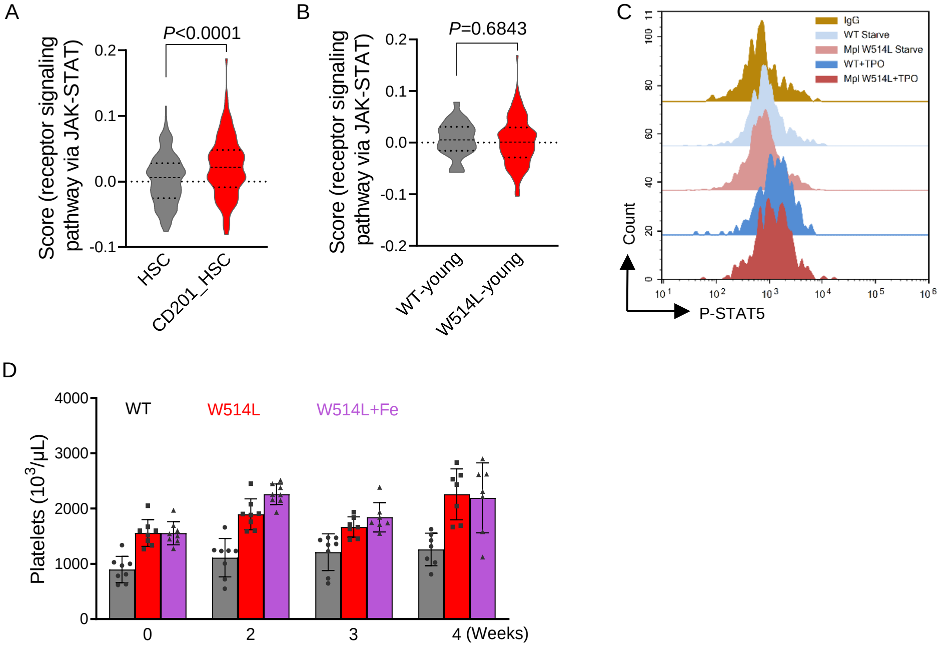

A
P<0.0001
Score (receptor signaling pathway via JAK-STAT)
0.2
0.1
0.0
-0.1
HSC
CD201_HSC
B
P=0.6843
Score (receptor signaling pathway via JAK-STAT)
0.2
0.1
0.0
-0.1
-0.2
WT-young
W514L-young
C
IgG
WT Starve
Mpl W514L Starve
WT+TPO
Mpl W514L+TPO
Count
P-STAT5
D
WT
W514L
W514L+Fe
Platelets (10³/μL)
4000
3000
2000
1000
0
0
2
3
4 (Weeks)
