# Supplementary figures and images for: Physiological MplW514L expression in hematopoietic stem cell causes an essential thrombocythemia and progressive myelofibrosis
Source: J Clin Invest. 2026 Apr 23;136(11):e199690. doi: 10.1172/JCI199690 (PMC13221233; doi:10.1172/JCI199690)

# Full unedited blots-Figure 1F

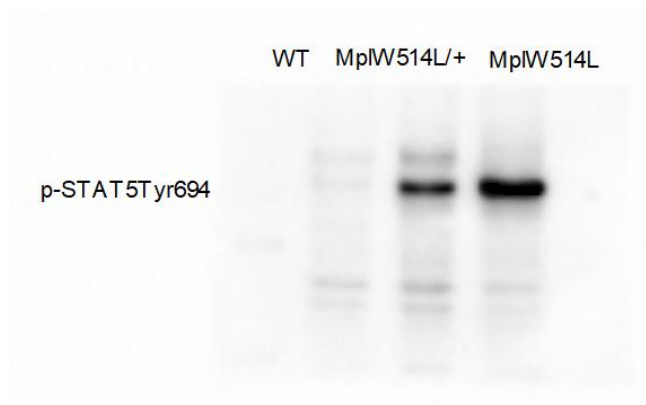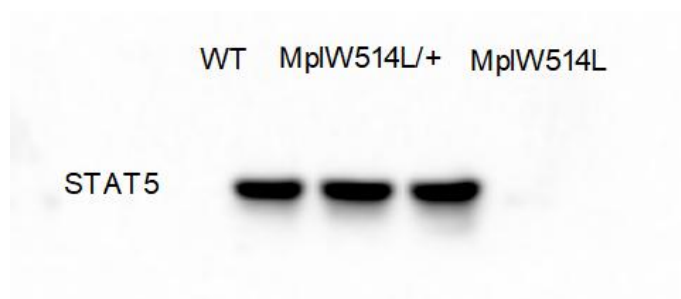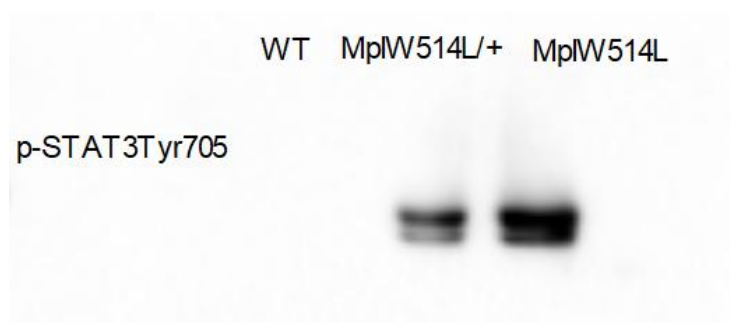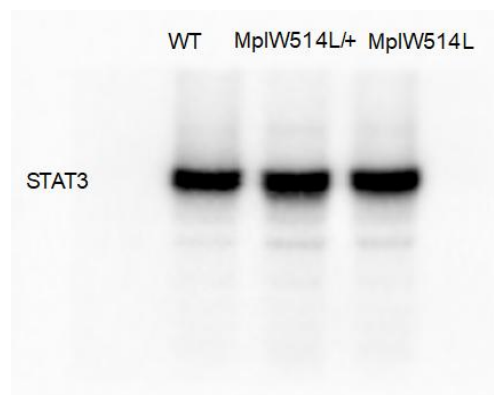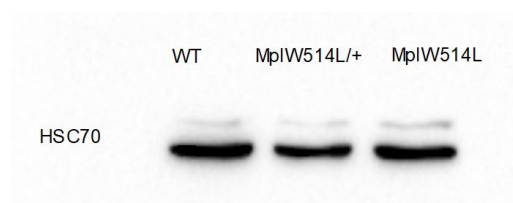

## Full unedited blots-Figure 7E

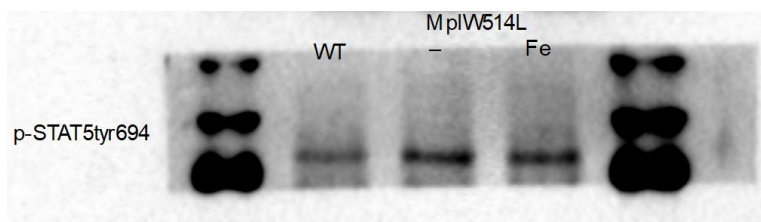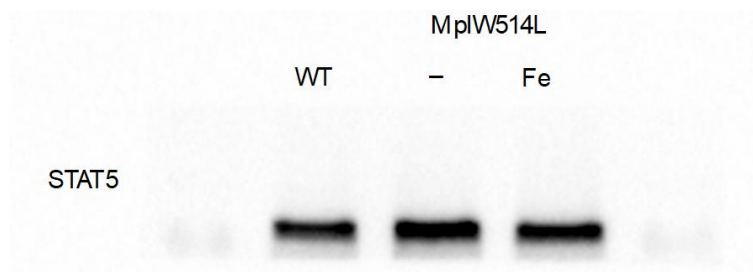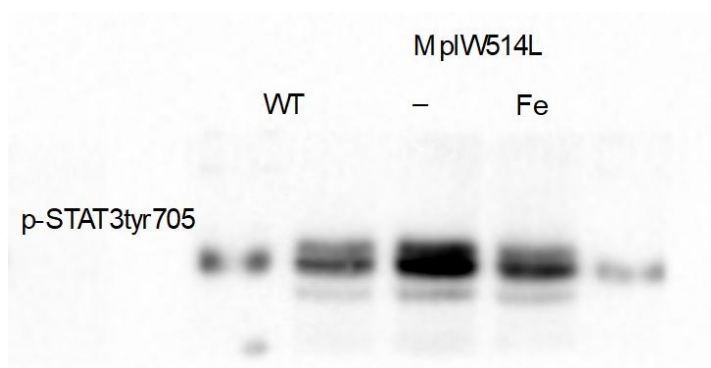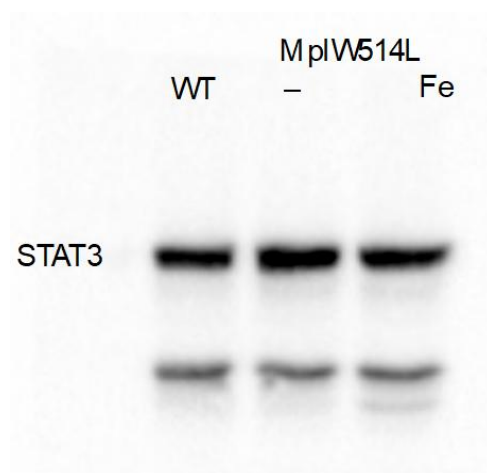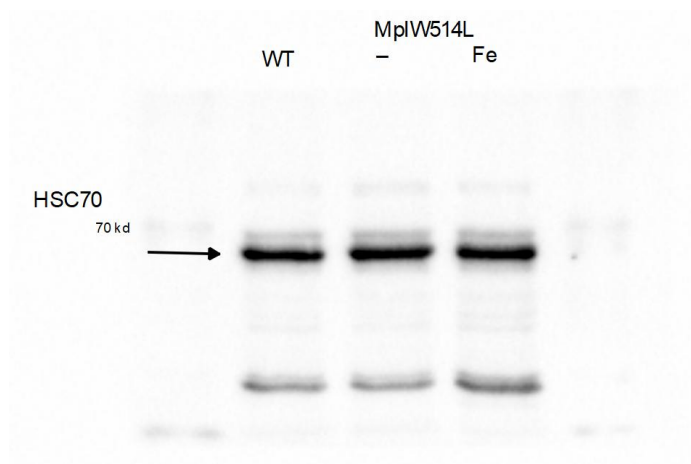

Supplement: Unedited blot and gel images [file jci-136-199690-s084.pdf]
